# Phenotypic Plasticity of the Drosophila Transcriptome

**DOI:** 10.1371/journal.pgen.1002593

**Published:** 2012-03-29

**Authors:** Shanshan Zhou, Terry G. Campbell, Eric A. Stone, Trudy F. C. Mackay, Robert R. H. Anholt

**Affiliations:** 1 Department of Biology, North Carolina State University, Raleigh, North Carolina, United States of America; 2 W. M. Keck Center for Behavioral Biology, North Carolina State University, Raleigh, North Carolina, United States of America; 3 Department of Genetics, North Carolina State University, Raleigh, North Carolina, United States of America; Stanford University School of Medicine, United States of America

## Abstract

Phenotypic plasticity is the ability of a single genotype to produce different phenotypes in response to changing environments. We assessed variation in genome-wide gene expression and four fitness-related phenotypes of an outbred *Drosophila melanogaster* population under 20 different physiological, social, nutritional, chemical, and physical environments; and we compared the phenotypically plastic transcripts to genetically variable transcripts in a single environment. The environmentally sensitive transcriptome consists of two transcript categories, which comprise ∼15% of expressed transcripts. Class I transcripts are genetically variable and associated with detoxification, metabolism, proteolysis, heat shock proteins, and transcriptional regulation. Class II transcripts have low genetic variance and show sexually dimorphic expression enriched for reproductive functions. Clustering analysis of Class I transcripts reveals a fragmented modular organization and distinct environmentally responsive transcriptional signatures for the four fitness-related traits. Our analysis suggests that a restricted environmentally responsive segment of the transcriptome preserves the balance between phenotypic plasticity and environmental canalization.

## Author Summary

Unlike Mendelian traits, where the genotype allows a direct prediction of the phenotype, predicting phenotypic values is not straightforward for complex traits, which arise from multiple segregating genes and their interactions with the environment. Here, a single genotype can often express different phenotypes in different environments. Such phenotypic plasticity is the counterpoint to “environmental canalization,” whereby genotypes produce the same phenotype in different environments. Whereas phenotypic plasticity allows organisms to respond rapidly to changing environments, environmental canalization buffers phenotypes against environmental perturbations. The balance between plasticity and robustness is crucial for optimal fitness, but the genetic basis for phenotypic plasticity is poorly defined. Here, we present the most comprehensive analysis to date of variation in genome-wide gene expression of an outbred *Drosophila melanogaster* population under 20 different environments. We find that a restricted environmentally responsive segment of the transcriptome (∼15%) preserves the balance between phenotypic plasticity and environmental canalization. Environmentally plastic transcripts can be grouped into two categories. Class I transcripts are genetically variable and associated with detoxification, metabolism, proteolysis, heat shock proteins, and transcriptional regulation. Class II transcripts have low genetic variance and show sexually dimorphic expression enriched for reproductive functions. Despite low genetic variance these transcripts evolve rapidly.

## Introduction

Phenotypic plasticity is the ability of a single genotype to give rise to different phenotypes in different environments [1]. Phenotypic plasticity is the counterpoint to environmental canalization [2]–[3], whereby genotypes produce the same phenotype in different environments. Phenotypic plasticity allows organisms to respond rapidly to changing environmental conditions without the time lag required for response to natural selection on segregating allelic variants and without the cost of selection, while environmental canalization buffers phenotypes against environmental perturbations. The balance between plasticity and robustness is thus crucial for optimal fitness [3]–[4] in variable environments, but the genetic basis for phenotypic plasticity has remained poorly defined.

Elucidating the genetic underpinnings of phenotypic plasticity (and its converse, environmental canalization) requires that we determine what fraction of the genome is environmentally sensitive, which genes respond to the same or different environmental perturbations and how expression of environmentally sensitive genes is correlated with plasticity of organismal phenotypes. It is also necessary to determine what the relationship is between genetic variance and phenotypic plasticity, whether the same genes affecting phenotypic plasticity for a trait also affect genetic variation for that trait, and whether environmentally plastic and environmentally robust genes evolve at different rates. Although previous studies have analyzed changes in gene expression under one or few different environmental or physiological conditions [5]–[11], the study presented here is the first comprehensive study that analyzes co-variation among environmentally responsive genes across a wide range of environments in a defined outbred population reconstructed from inbred lines with documented expression profiles, enabling us to compare genotypic and environmental variation.

We examined phenotypic plasticity in genome-wide gene expression and four organismal phenotypes related to reproductive fitness in a population generated by crossing 40 wild-derived inbred *D. melanogaster* lines [12]. The majority of the transcriptome shows robust expression across a range of environmental challenges, including different nutritional rearing conditions, physical stress conditions, chemical exposures, social crowding during larval or adult stages, and aging. Approximately 15% of transcripts are phenotypically plastic. By comparing genotypic variation among the original 40 wild-derived inbred lines under standard growth conditions, documented earlier [12], with environmental variation of transcript abundance levels in the reconstituted outbred population, we were able to discriminate two distinct classes of environmentally responsive transcripts, which we have designated Class I and Class II transcripts.

## Results

### Phenotypic Plasticity of the Transcriptome

To identify phenotypically plastic and environmentally canalized transcripts, we assessed genome-wide gene expression of flies exposed to 20 treatments, including a control treatment of mated flies reared under standard conditions, and different nutrient or drug supplements, exposure to different physical and social environments, and maintenance at different reproductive states. Of the 18,800 transcripts represented on the microarray, 14,400 (76.6%) generated signal intensities above background under at least one treatment, similar to the proportion of the transcriptome detected in a previous study, in which transcript profiles were obtained separately for the 40 individual genotypes that gave rise to our outbred population [12]. Analysis of variance of microarray intensity signals across all 20 rearing conditions revealed 1,249 transcripts that showed a significant treatment effect (8.7%), 6,745 transcripts that showed a sex effect (46.8%), and 200 transcripts with a significant treatment by sex interaction term (1.4%) at a false discovery rate of 0.05. Thus, the majority of the transcriptome is remarkably robust and buffered against diverse environmental challenges.

We refer to the 1,249 transcripts exhibiting phenotypic plasticity as quantified by the significant treatment term in the ANOVAs as Class I transcripts. To simplify statistical analyses and maintain optimum power we excluded 166 Class I transcripts that also had significant treatment by sex interaction terms, giving 1,133 phenotypically plastic transcripts for further analyses (Table S1). We grouped these transcripts according to their relative expression levels across the 20 conditions (Figure S1). The highest relative gene expression levels were observed in flies exposed to low temperature, dopamine, nicotine or high sugar, and the lowest relative levels in flies exposed to heat shock or grown on high yeast medium. Surprisingly, overall relative gene expression levels are either uniformly higher or lower (more than 70% show higher abundance levels than the median) across the 20 conditions; however, starvation stress resistance, aging, larval crowding and exposure to high temperature, result in substantial variation among relative expression levels (Figure S1).

**Figure 1:**
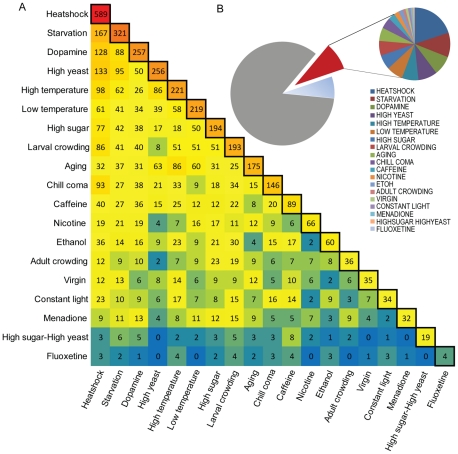
Class I phenotypically plastic transcripts across 19 treatments compared to the standard rearing condition. (A) Transcripts with differential expression levels under different experimental treatments compared to their expression under the standard condition. The blue-red color scale accentuates increasing numbers of transcripts. Pair-wise comparisons indicate the number of overlapping transcripts with differential expression under two conditions. (B) Proportion of phenotypically plastic transcripts. The gray area of the pie chart indicates the proportion of the transcriptome that does not undergo altered expression under 20 different environmental conditions. The red slice indicates the proportion of genes that show differential expression compared to their expression under the standard rearing condition, and the pie-chart insert shows the proportion of those genes that are affected by each of the 19 treatments. Treatments are ordered clockwise from the largest pie slice. Transcripts are identified in Table S2. The blue slice indicates the Class II phenotypically plastic sensitive transcripts.

To further examine the relationship between gene expression and environmental exposure, we compared transcript abundance levels of the Class I transcripts under the different treatments to the standard rearing condition with *post hoc* least square difference (LSD) tests (*p*<0.05; Table S2). Heat shock has the greatest impact on gene expression (589 transcripts), whereas fluoxetine changes expression of only four transcripts (Figure 1A). Many transcripts show altered expression under multiple treatments; for example, 167 transcripts show altered expression both after heat shock and exposure to starvation (Figure 1A). The majority of transcripts do not undergo significant changes compared to the standard condition (Figure 1B).

**Figure 2:**
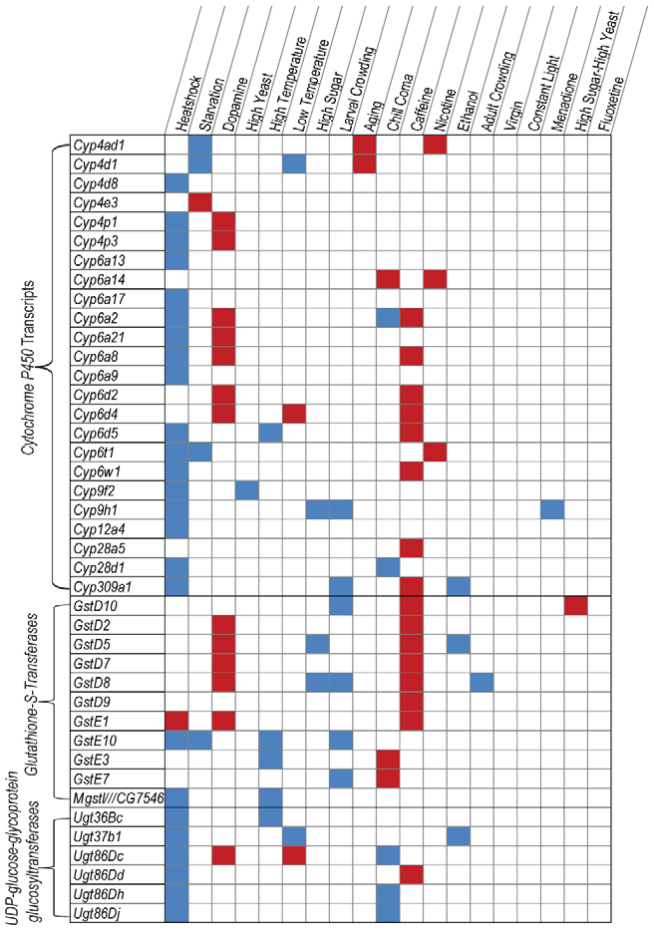
Class I phenotypically plastic transcripts associated with xenobiotic metabolism. Up-regulation or down-regulation of members of the cytochrome P450, glutathione-S-transferase and UDP-glucose-glycoprotein glucosyltransferase families under different treatments compared to the standard growth condition is indicated by red and blue boxes, respectively.

Among the 1,133 Class I transcripts, 691 are computationally predicted with unknown function, 14 probe sets correspond to intergenic regions, non-coding RNAs and transposons, and 428 are annotated. The transcripts that show altered expression after heat shock include 13 *Heat shock proteins* (*Hsp*s), 60 proteases, 17 members of the cytochrome P450 family (*Cyp*s), two glutathione-S-transferases (*Gst*s), six UDP-glucose glycoprotein glycosyltransferases (*Ugt*s), and six immune-induced molecules (*IM*) (Figure 2, Table S2; Figure S2). A variety of additional transcripts in diverse gene ontology (GO) categories also respond to environmental challenges (Table S3). Nine of the 13 *Hsp*s upregulated after heat shock are also upregulated after induction of chill coma (Figure S2). The abundance of heat shock proteins and proteases encoded by environmentally sensitive genes reflect mechanisms for environmental adaptation of the proteome. Heat shock proteins may offer protection for nascent polypeptides under adverse temperature conditions, while one function of environmentally sensitive proteases may be to facilitate *de novo* protein synthesis by providing a pool of amino acids through degradation of dispensable proteins.

### Modules of Phenotypically Plastic Transcripts

**Figure 3:**
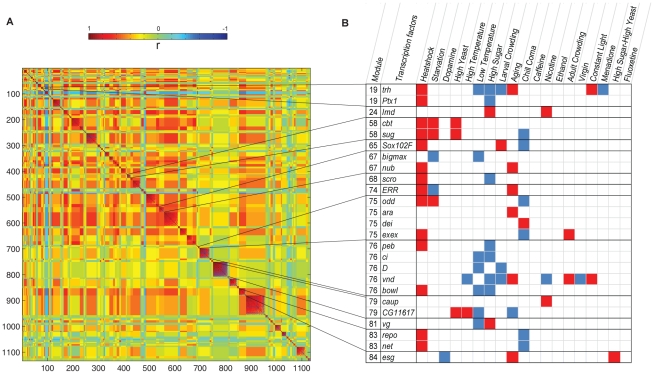
Modular organization of the Class I phenotypically plastic transcriptome. (A) Partitioning of the 1,133 Class I phenotypically plastic , shown in Figure S1 and Table S1, into 103 covariant modules by MMC [13]. The modules populate the diagonal and are ordered by decreasing strength from the upper left to the lower right. Note the pervasiveness of cross-module correlations. The *Cyp* transcripts are distributed across multiple modules, which may reflect their specialized functions. However, *Cyp6a21*, *Cyp6a2*, and *Cyp6d5*, along with *Ugt86Dd*, group in module 18; *Cyp6w1* and *Cyp6a8*, co-vary in module 49; *Cyp6a17*, *Cyp12a4*, and *Cyp9f2* cluster in module 68; and, *Cyp4d1* and *Cyp4ad1*, both associated with aging, co-vary in module 101. *GstD2* and *GstD9* cluster with *Cyp6d2* in module 45. (B) Class I phenotypically plastic transcription factors. The diagram lists 25 phenotypically plastic transcripts that encode transcriptional regulators and the black lines connect these transcription factors to the modules that contain them in panel A (only modules that contain multiple transcription factors are indicated). Red boxes and blue boxes designate up-regulation and down-regulation, respectively, for each treatment compared to the standard growth condition.

We asked to what extent expression patterns of phenotypically plastic transcripts are co-regulated across environmental treatments. A previous study on the 40 inbred lines from which our outbred population is derived demonstrated that the genetically variable transcriptome (10,096 transcripts) is highly inter-correlated and can be subdivided into 241 co-regulated modules [12], identified by modulated modularity clustering (MMC) [12]–[13]. Here, we used MMC to identify transcripts that covary across different treatments (Figure 3A, Table S1). This analysis partitioned the 1,133 Class I transcripts into 103 small, but highly correlated transcriptional modules (the average absolute correlation coefficient, |*r*|, within modules is at least 0.56). Extensive cross-module correlations are also evident. Negative correlations are rare, in agreement with the overall uniform up- or down-regulation of transcripts (Figure S1). All seven *IM* transcripts group together in module 71. A putative *IM*, *CG15065*, which is genetically correlated with *IM1* and *IM3* [12], is also contained in this module (Figure S2). These results show that changes in environmental conditions can cause fragmentation of the highly intercorrelated structure of the transcriptome observed under the standard growth condition [12].

### Phenotypically Plastic Transcription Factors

**Figure 4:**
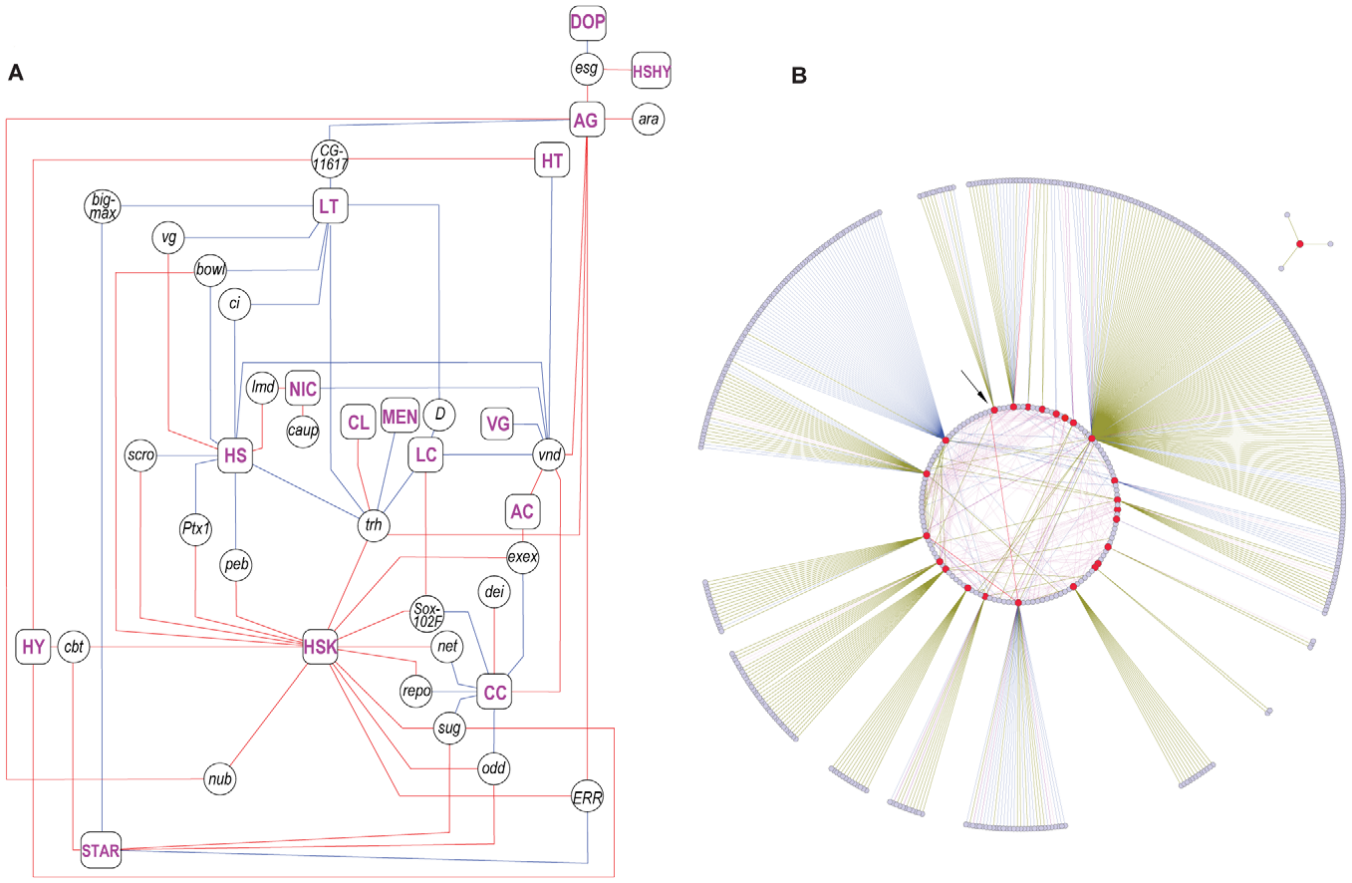
Class I phenotypically plastic transcription factors. (A) Diagram of the relationship between transcription factor regulation and rearing conditions. The 25 environmentally sensitive transcription factor transcripts are shown in circles and 16 treatment conditions are shown in magenta font in rectangular boxes. Red and blue lines designate up- and down- regulation of the transcription factor, respectively, under different treatments. Designations are: HSK, heatshock; STARV, starvation; DOP, dopamine (DOP); HY, high yeast; HT, high temperature; LT, low temperature; HS, high sugar; LC, larval crowding; AG, aging; CC, chill coma; NIC, nicotine: AC, adult crowding; VG, virgin; CL, constant light; MEN, menadione; HSHY, high sugar-high yeast. (B) Interaction networks of phenotypically plastic transcription factors. Interaction networks of the 25 transcription factors (red nodes) were analyzed through the *Drosophila* Interaction Database (DroID) [36], [37]. The diagram shows protein-protein interactions (green edges), genetic interactions (blue edges), protein-DNA interactions (red edges), interactions that involve both protein-protein, genetic and/or protein-DNA interactions (magenta edges), and interactions with miRNAs (pink edges). Interactions with single transcription factors are shown around the periphery, whereas multiple interactions between transcription factors and interactions between genes (or micro RNAs) and multiple transcription factors are shown inside the circle. Transcription factors are clockwise starting from the arrow: *bowl, ara, caup, cbt, exex, CG1617, trh, ci, net, ERR, lmd, repo, ptx1, scro, Sox102F, sug, vg, D, vnd, dei, odd, nub, esg,* and *peb*. Only three interactions are documented for *bigmax*, shown as a separate diagram.

What are the cellular mechanisms that regulate transcriptional responses to environmental changes? As a first step to investigating how environmental stimuli may influence transcriptional regulation, we asked which transcription factors show altered expression under the different environmental conditions. Among the Class I transcripts, we identified 26 transcripts that encode transcriptional regulators, of which 25 were differentially expressed relative to the standard growth condition (Figure 3B, Figure 4A). Many of these transcription factors occur together in the same transcriptional modules (Figure 3B). The complexity of the interrelationships between transcript abundance levels of different transcription factors under different growth conditions is further illustrated in Figure 4A.

Each transcription factor can exert wide-ranging effects on networks of interacting genes that include other regulatory genes, non-regulatory genes and miRNAs [14]–[15] (Figure 4B). Direct genetic, protein-protein and gene-protein interactions among these phenotypically plastic transcription factors appear compartmentalized, with little overlap between interacting components related to each transcription factor. In contrast, there is an extensive network of interactions among microRNAs and different transcription factors (Figure 4B). Extensive interactions of transcription factors with miRNAs suggest that these may also contribute to phenotypic plasticity of the transcriptome [14]–[15]. In addition, seven long non-coding RNAs and unannotated intergenic regions are phenotypically plastic, and genes that encode several phenotypically plastic transcripts contain overlapping or flanking sequences for short non-coding RNAs (Table S1). Finally, we note that predicted transcripts of unknown function may also play a regulatory role. In addition, transcriptional regulators that themselves show no change in gene expression may be regulated by phenotypically plastic post-translational modifications.

### Phenotypic Plasticity of Organismal Phenotypes

We next asked to what extent the phenotypic plasticity in gene expression is associated with phenotypic plasticity of organismal phenotypes. We assessed phenotypic plasticity of four fitness-related phenotypes: development time, lifespan, starvation stress resistance, and chill coma recovery time.

**Figure 5:**
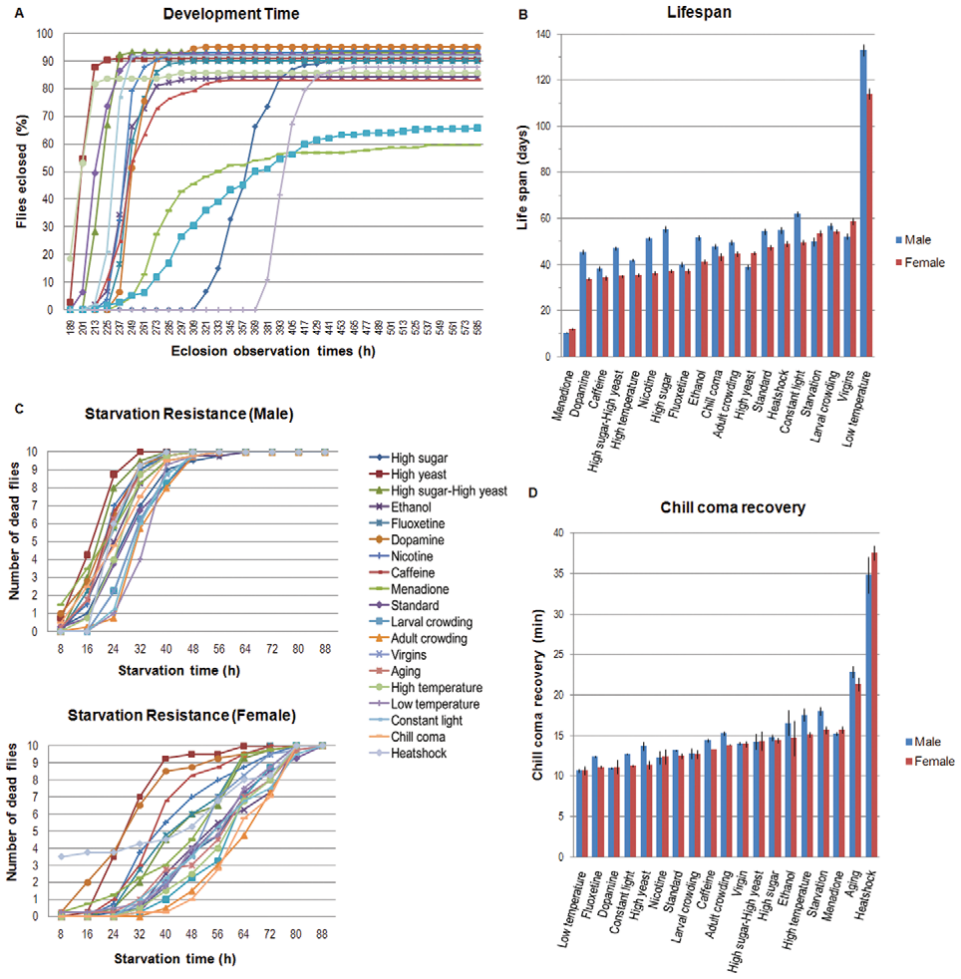
Variation for organismal phenotypes under various treatment conditions. (A) Development latency. Development time was assessed under 14 conditions (adult stage treatments were excluded). The X-axis indicates eclosion times after egg collection for sexes pooled. (B) Lifespan. Average survival times were measured for flies reared under 19 different experimental treatments. (C) Starvation stress resistance. The number of dead flies was counted at different times following food deprivation under 19 different treatment conditions. (D) Chill coma recovery time. Average recovery times from chill coma were measured for flies reared under 19 different experimental treatments. Blue and red bars indicate males and females, respectively. Error bars, s.e.m.

Development is exquisitely sensitive to environmental conditions [16]–[17] (Figure 5A) and is accelerated when flies are grown on medium supplemented with high yeast, and delayed when the medium is supplemented with high sugar. When grown on both high sugar and high yeast, development time is identical to that under the standard growth condition. Growth at 28°C also accelerates development, but reduces survival, whereas growth at 18°C delays development about 2-fold. Larval crowding and exposure to the chemical oxidative stress agent menadione sodium bisulfite results in delayed development along with reduced survival. Medium supplemented with 10% ethanol has a small effect on development time and survival. All other treatments lead to slower development compared to the standard condition.

In addition to prolonging development, growth at 18°C results in a two-fold increase in lifespan (Figure 5B). Furthermore, virgin females live longer than mated females, as expected [18]. When reared under standard conditions, subsequent food deprivation allows females to survive twice as long as males (Figure 5C). Survival curves indicate a trend towards longer survival times for both sexes when flies are maintained at high density, either as larvae or adults, or at 18°C. Exposure to chill coma tends to increase survival time during subsequent food deprivation in females (Figure 5C). Previous exposure to heat shock extends chill coma recovery time. Recovery is also delayed as a result of aging, growth at 28°C, previous exposure to starvation stress, growth on ethanol, menadione sodium bisulfite, or high sugar-supplemented medium, and maintenance at high density as adults (Figure 5D).

Whereas caloric restriction extends lifespan [19]–[20], a single 24 h food deprivation period does not affect lifespan. The increase in starvation stress resistance following chill coma recovery may be due to slowing of intermediary metabolism during chill coma and, consequently, preservation of metabolic energy.

### Phenotypic Plasticity of Transcripts Correlated with Organismal Phenotypes

**Figure 6:**
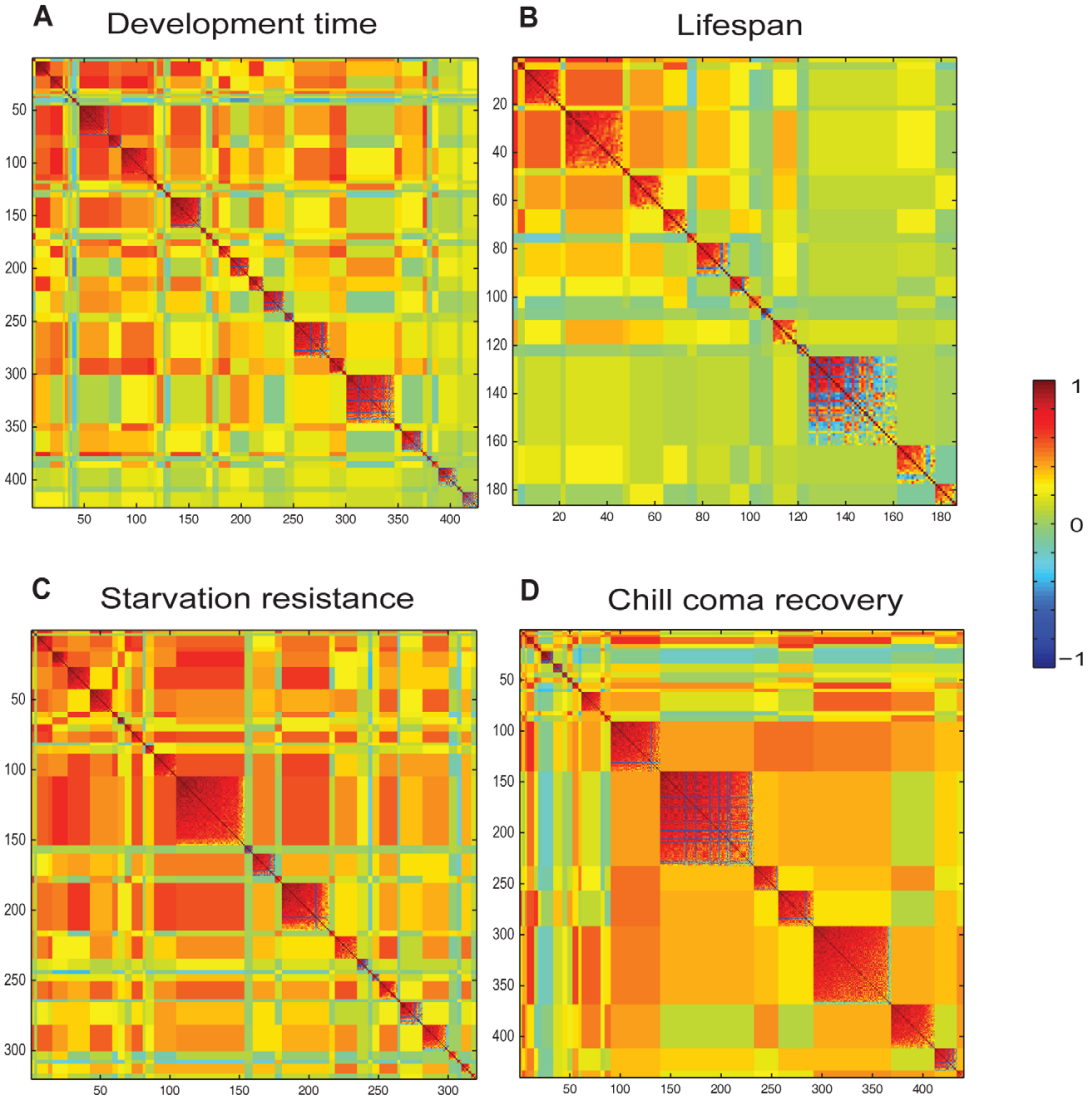
Partitioning of correlated Class I phenotypically plastic transcripts associated with organismal phenotypes across different rearing conditions by MMC [13]. (A) Clustering of 426 genes significantly associated with variation in developmental latency into 116 modules. (B) Clustering of 186 genes significantly associated with variation in lifespan into 16 modules. (C) Clustering of 320 genes significantly associated with variation in starvation resistance into 32 modules. (D) Clustering of 440 genes significantly associated with variation in chill coma recovery into 23 modules. The modules populate the diagonal and are ordered by decreasing strength from the upper left to the lower right. Transcripts associated with the four phenotypes are indicated in Tables S4, S5, S6, S7.

We used regression to identify Class I transcripts associated with variation in organismal phenotypes across the 20 environmental conditions, and MMC to construct environmentally correlated modules [13] of these transcripts (Figure 6). Phenotypic plasticity in development time is associated with 426 transcripts, of which 411 cluster into 36 highly correlated (average |*r*|>0.5) modules (Figure 6A). Similarly, phenotypic plasticity in lifespan, starvation stress resistance and chill coma recovery is associated with, respectively, 186, 320 and 440 transcripts, which cluster into 12, 30 and 23 highly correlated (average |*r*|>0.5) modules, respectively (Figure 6B–6D, Tables S4, S5, S6, S7). Modules associated with each trait show high degrees of inter-correlation, and there is evidence for cross-module clustering, indicating hierarchical co-regulation of the Class I plastic genes (Figure 6). We found little overlap (∼3%) between transcripts associated with genetic variation in lifespan, starvation resistance, and chill coma recovery under the standard growth condition [12] and transcripts associated with phenotypic plasticity of these traits. Since 1,125 of the 1,133 (99.3%) Class I transcripts are also genetically variable, the lack of concordance between the association with genetic and environmental phenotypic variation cannot be attributed to the trivial explanation that the genetically variable and phenotypically plastic transcripts do not overlap.

Some modules associated with different organismal phenotypes are enriched for common transcripts, indicating pleiotropy for phenotypic plasticity (Figure S3). For example, the chill coma recovery module 17 contains the same transcripts as modules associated with phenotypic plasticity in development time and in starvation stress resistance. Whereas pleiotropy at the level of covariant transcriptional modules is prominent between chill coma recovery, starvation stress resistance and development time, it is sparser between lifespan and the other traits (Figure S3).

In summary, clustering analysis of Class I transcripts reveals a fragmented modular organization and distinct environmentally-responsive transcriptional signatures for the four fitness-related traits.

### Class II Phenotypically Plastic Transcripts

**Figure 7:**
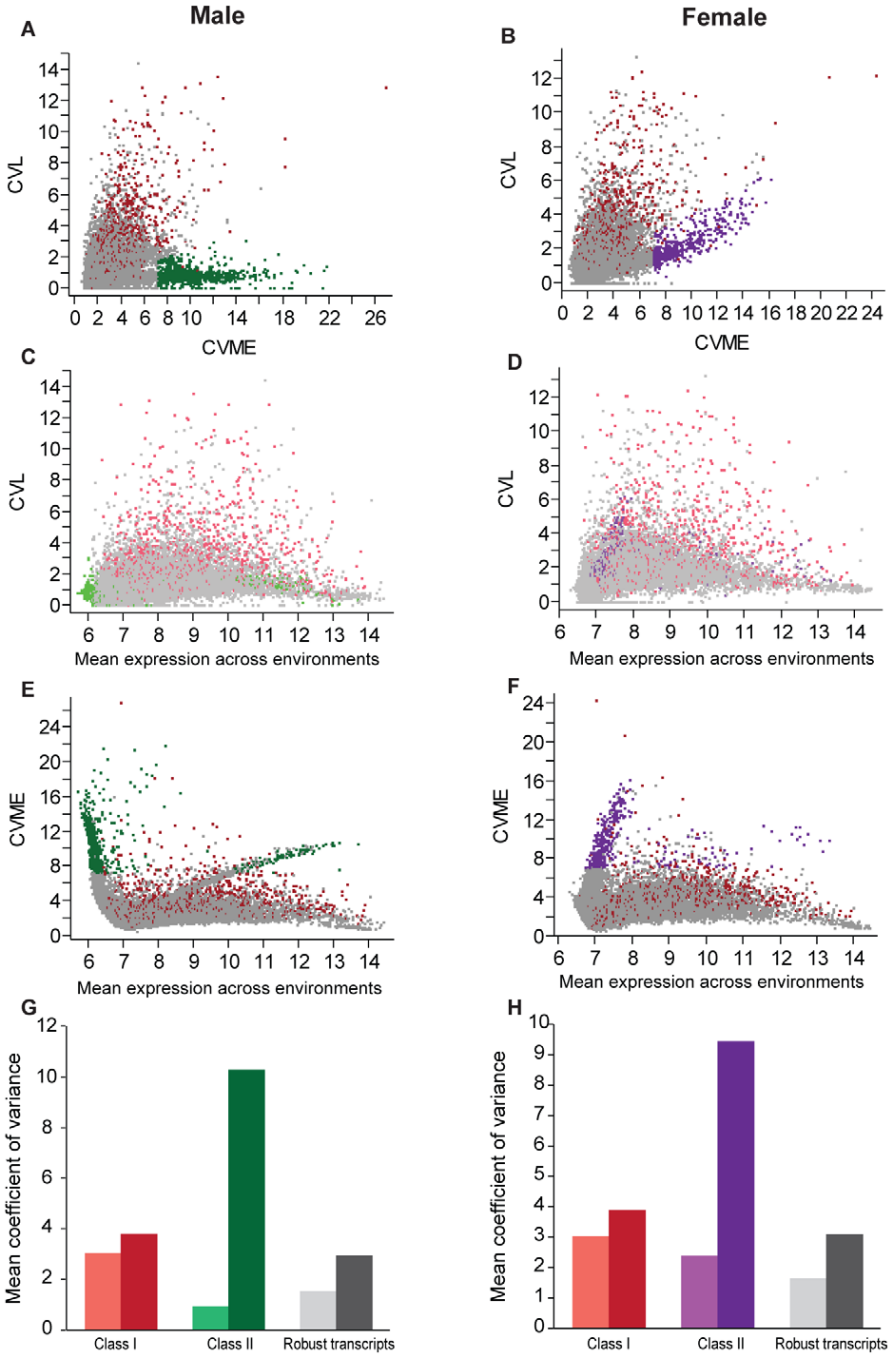
Variance analysis and classification of phenotypically plastic and robust transcripts. (A, B) Relationships between coefficients of genetic variance of inbred lines (CVL) and coefficients of macroenvironmental variance across treatments (CVME) in males (A) and females (B). (C, D) Distributions of coefficients of genetic variance of inbred lines with respect to mean transcript expression levels over all environments in males (C) and females (D). (E, F) Correlation structures between coefficients of macroenvironmental variance and mean transcript expression levels over all environments in males (E) and females (F). Class II transcripts explain the majority of the correlation structure. Red dots indicate Class I transcripts, green and purple dots indicate Class II transcripts in males (A, C, E) and females (B, D, F), and grey dots indicate robust transcripts. (G,H) Average coefficients of genetic variance across inbred lines (light shades) and macroenvironmental variance across treatments (dark shades) of each transcript class in males (G) and females (H).

To assess the relationship between genetic variation and phenotypic plasticity, we compared the previously reported genetic variance and micro-environmental variation (within-line variation) across the 40 inbred lines [12] from which our outbred population was derived, reared under the standard growth condition, with the variation in phenotypic plasticity (macro-environmental variation) and within-treatment variation of the same transcripts in the outbred population. We quantified genetic variation as the coefficient of variation among lines (CVL) and variation in phenotypic plasticity as the coefficient of macroenvironmental variance (CVME). We found a strong correlation between genetic variation and variation in phenotypic plasticity for Class I transcripts in both sexes (Figure 7A, 7B).

This comparison revealed an additional group of 982 environmentally sensitive transcripts with high macroenvironmental variation, but low genetic variance (Figure 7A, 7B, Figure S4, Table S1). These phenotypically plastic transcripts, which we designate as Class II, were not identified by our initial analysis due to high within-treatment environmental variation (quantified as the coefficient of variation within environments, CVEW, Figure S5A–S5D). Phenotypic plasticity for Class II transcripts was mostly sexually dimorphic, with 230 transcripts specific to females, 560 specific to males, and 192 in common for both sexes (Table S1). Class I transcripts have greater genetic variation for both sexes than environmentally robust transcripts, which are relatively stably expressed both across genotypes and treatments (Figure 7G, 7H, Figure S4C, S4D). In males the average genetic variance of Class II transcripts is lower than both Class I and robust transcripts (Figure 7G, Figure S4A), while in females the average genetic variance of Class II transcripts is lower than the Class I but higher than the robust transcripts (Figure 7H, Figure S4B). In contrast to the genetic variance, the average macroenvironmental variation across treatments of Class II transcripts is ∼two-fold greater than that of Class I transcripts for both sexes (Figure 7G, 7H, Figure S4C, S4D ).

There is little correlation between the level of genetic (Figure 7C, 7D) and macroenvironmental (Figure 7E, 7F) variation with the mean level of gene expression across all environments for Class I and robust transcripts. However, the macroenvironmental variance (Figure 7E, 7F) as well as the variance in gene expression within treatments (Figure S5A–S5D) and within inbred lines (Figure S5E, S5F) are correlated with the mean expression across treatments. To exclude the possibility that this observation is an artifact due to array quality, we examined the correlation between the previously published mean expression levels of transcripts across the 40 inbred lines [12] and the mean expression level in the outbred population across conditions. Transcript means were highly correlated between the two experiments (*r* = 0.960 and *r* = 0.963, for females and males, respectively; Figure S5G, S5H).

Since Class II transcripts exhibited sexual dimorphism in phenotypic plasticity, we evaluated the relationship between sexual dimorphism in mean gene expression across all 20 environments, and sexual dimorphism for phenotypic plasticity, for Class I and Class II transcripts as well as a sample of robust transcripts (Figure S6). We found a clear inverse relationship between sexual dimorphism for mean expression and sexual dimorphism for the variance in expression across environments for Class I transcripts, but not the other categories. Female-biased Class II genes for mean expression are male-biased for plasticity in expression, and *vice versa*.

Class II phenotypically plastic transcripts can be further classified into high and low expression categories. Highly expressed transcripts in females overlap transcripts with low expression in males, and GO analysis shows that these 19 transcripts encode yolk proteins and chorion proteins and are enriched for oogenesis and sexual reproduction (Table S8). Similarly, transcripts with low expression in females overlap transcripts with high expression in males, and GO analysis shows that these 162 transcripts encode male-specific proteins, accessory gland proteins and hormones which affect mating and post-mating behaviors (Table S9). Further GO analyses indicate that female-specific Class II transcripts are enriched in mitochondria- and muscle-related functions, whereas male-specific transcripts are enriched in functions of cuticular structure and DNA replication in meiosis (Tables S10 and S11). Enrichment of Class II transcripts for reproductive functions suggests that the high environmental responsiveness of these transcripts may protect reproductive fitness.

### Conservation of Phenotypically Plastic Genes

**Figure 8:**
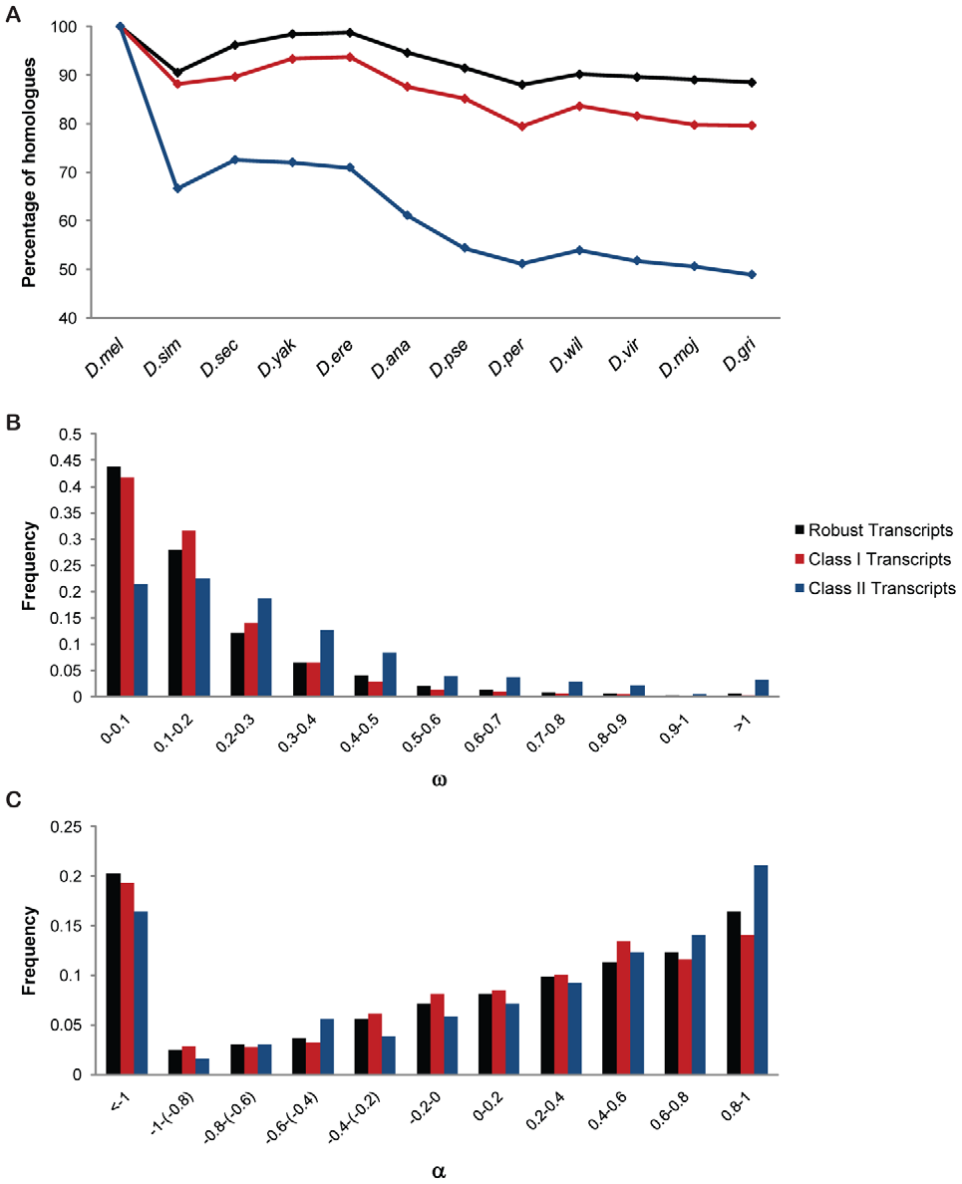
Cross species analyses of transcript classes. (A) Percentage of homologues across 12 *Drosophila* species. (B) Distribution of ω (d_N_/d_S_), using *D.yakuba* as outgroup species. Both Class I and Class II transcripts have significantly different distributions of ω from that of the robust transcripts (G = 22.95, *p* = 0.01, and G = 261.52, *p*<0.00001, respectively). (C) Distribution of α (1−(P*_i neutral_*/*P_0_*)(*D_0_*/*D_i_*)), using *D.yakuba* as outgroup species. Class II transcripts have significantly different distributions of α from that of the robust transcripts (G = 28.34, *p* = 0.0015).

To assess to what extent phenotypically plastic genes are evolutionarily conserved compared to the rest of the genome, we looked at the percentage of homologues across 12 *Drosophila* species, the ratio of non-synonymous to synonymous substitutions (ω) and fraction of adaptive fixations (α) using *D. yakuba* as outgroup [21]–[23] (Figure 8, Figure S7). Class I genes are less conserved across the *Drosophila* clade and have less constraints under selection than the environmentally robust genome. Previously, we documented plasticity of the *Drosophila* chemoreceptor repertoire [24]. Like chemoreceptor genes, many of the Class I transcripts also belong to rapidly evolving multigene families (Figure 8). Such rapid evolution may involve gene duplication and subfunctionalization, as is evident within the *Cyp* gene family [25]–[29].

Class II genes show an even faster rate of evolution compared to robust transcripts with a significantly higher proportion of positively selected sites, as evident from the distributions of ω and α (Figure 8). Thus, these phenotypically plastic genes appear especially responsive to natural selection.

## Discussion

Genome-wide transcriptional analysis of flies reared under 20 environmental conditions shows that ∼15% of the transcriptome exhibits phenotypic plasticity, while the rest is environmentally canalized. Logistical and economic constraints have limited this initial investigation to whole flies. We surveyed the FlyAtlas database [30] and found that transcripts associated with all organismal phenotypes are generally expressed in multiple, but not all tissues (Figure S8). In future studies it would be of interest to examine directly tissue-specific environmental modulation of expression of phenotypically plastic transcripts. Since we only examined adult flies, we could not detect transcripts that may show environmental plasticity at different developmental stages. Furthermore, although we provided a comprehensive analysis of the transcriptional response to a vast variety of conditions and treatments, additional treatments, *e.g.* different chemical exposures or sleep deprivation, might reveal additional features of the phenotypically plastic transcriptome. However, results from previous studies on genome-wide transcriptional responses to environmental and physiological changes in *Drosophila* are in line with our observations [6], [8], [11]. A previous study that examined phenotypic plasticity of the transcriptome during aging and upon exposure to paraquat-induced oxidative stress reported altered regulation of *Hsp26*, several metabolic enzymes, and glutathione-S-transferases [6]. Genome-wide transcriptional profiling during aging under conditions of caloric restriction also showed plastic responses of genes associated with xenobiotic defense and reproduction [8]. In addition, phenotypically plastic transcripts associated with xenobiotic defense, metabolism and chitin biosynthesis have been identified in *Drosophila* populations from tropical and temperate zones in Eastern Australia in response to temperature [11]. The analysis presented here is the most comprehensive study of phenotypic plasticity to date, which capitalizes on the unique properties of an outbred population reconstructed from well characterized inbred wild-derived lines, which enabled us to discriminate two classes of plastic transcripts.

Class I transcripts are not only phenotypically plastic, but are more genetically variable and evolve more rapidly than the rest of the transcriptome. Class I transcripts are enriched in functions of detoxification, metabolism, proteolysis and heat shock proteins. Class I transcripts also encode gene products of unknown function, including non-coding RNAs, which may contribute to modulation of chromatin structure and transcriptional regulation. The coupling of high genetic variation within a population and rapid evolution suggests interesting evolutionary forces acting on these genes.

Class II transcripts have low genetic variance for mean expression levels, but greater environmental variation in transcript abundance, and are even more rapidly evolving than Class I transcripts. It is tempting to speculate that reduced genetic variation for these transcripts within a population is the consequence of selection favoring genotypes with high phenotypic plasticity within each species, but with variable selection pressures across species [31]. Under this hypothesis genotypes with high transcriptional plasticity would be fixed within a species but divergent across species, which implies there is genetic variation in phenotypic plasticity on which selection acts. We note, however, that non-additive effects may confound inferences based on comparing an outbred population in many environments with inbred genotypes in one environment.

Two models of the genetic basis of phenotypic plasticity have been postulated [32]. Under the ‘allelic sensitivity’ model, the same alleles affect the mean value of a phenotype and its plastic response to environmental variation. Under the ‘gene regulation’ model, plasticity is a trait in itself, under the control of regulatory loci which modulate the expression of other genes in different environments. Our comparison of transcripts for phenotypic plasticity in an outbred population with genetically variable transcripts among the 40 inbred genotypes from which the outbred population was derived support the gene regulation hypothesis. We found no more overlap than expected by chance between transcripts associated with the mean and plasticity of four fitness-related traits. Class II phenotypically plastic transcripts are highly sexually dimorphic, but male-biased plastic transcripts are associated with female-biased mean expression levels, and *vice versa*, again suggesting an uncoupling between the mean and macroenvironmental variance. The Class I plastic transcripts cluster into modules of highly correlated transcripts, with a high degree of correlation across modules, further implicating co-regulation of plastic responses to environmental variation. Whereas this initial comprehensive survey of phenotypic plasticity is necessarily largely descriptive, it provides a foundation for future studies aimed at testing mechanisms that link environmental inputs to alterations in gene expression. It is likely that the environmentally plastic transcriptional regulators which we identified (Figure 4) will play a role in mediating these responses. Furthermore, since we did not consider the effects of DNA sequence variants on phenotypic plasticity, co-regulated modules of phenotypically plastic transcripts are undirected. Deriving causal transcriptional networks for genetic variation in phenotypic plasticity requires superimposing genetic variation [33]. The recent availability of whole genome sequences of the wild-derived inbred lines from the Raleigh population will enable such analyses in the future [23].

## Materials and Methods

### Fly Rearing

We generated a synthetic outbred population by round-robin crossing of 40 wild derived inbred lines of the *Drosophila* Genetic Reference Panel (DGRP) [12], [23], followed by random mating for over 47 generations. For age-synchronization, we randomly collected 1000 females and 1000 males and allowed oviposition for 12 h on grape agar plates supplemented with yeast paste. Unless indicated otherwise, 55 eggs were collected and subjected to different treatments throughout development. The standard rearing condition (cornmeal (65 g/L) -molasses (45 ml/L) -yeast (13 g/L)- agar medium at 25°C, 60–75% relative humidity and a 12 h light-dark cycle) resulted in hatching of ∼50 larvae. Adults were collected immediately after eclosion, and placed at a density of 25 females and 25 males under the desired condition. Flies were transferred onto fresh medium every two days.

### Experimental Treatments

For nutritional and pharmacological treatments, flies were reared on standard medium supplemented with 225 ml/L molasses (‘high sugar’), 65 g/L yeast (‘high yeast’), 225 ml/L molasses and 65 g/L yeast (‘high sugar-high yeast’), 10% (v/v) ethanol, 200 µM fluoxetine hydrochloride, 47 mM dopamine, 1 mM nicotine, 2 mM caffeine or 4 mM menadione sodium bisulfite. Different physical environments included constant light, 28°C (‘high temperature’), 18°C (‘low temperature’), and exposure to different stresses, including heat shock (37°C for 1 h; 1 h recovery prior to RNA extraction), chill coma (3 h on ice; 1 h recovery prior to RNA extraction), and 24 h starvation. Different social environments included larval crowding (300 eggs/vial) and adult crowding (80 females and 80 males were pooled in each vial immediately after eclosion). To compare mated with non-mated flies, 50 single sex virgins were reared separately. Flies reared under standard conditions were mated. Aged flies were 30 days old.

### Whole-Genome Transcript Analysis

We used Affymetrix Drosophila 2.0 arrays to assess whole genome transcriptional profiles. Males and females (3–5 days old) were collected between 1:00–3:00 pm by aspiration and immediately frozen on dry ice. RNA was extracted from three independent samples (30 flies/sex/condition), and 10 µg of biotinylated, fragmented cRNA was hybridized to each microarray. RNA extraction, labeling and hybridization were randomized across samples. Raw data were log_2_ transformed and normalized across sexes and conditions using a median standardization. For each probe set, we used the median log_2_ signal intensity as the measurement of expression. We used negative control probe sets to estimate background intensity. Probe sets with hybridization intensities below background under all different treatment conditions were removed from the analysis. We did not correct for probe mismatches due to segregating polymorphisms in the reconstituted outbred population, because (1) the average hybridization bias will be identical across all environmental conditions, and; (2) only about 3,000 single feature polymorphisms (SFPs) were identified among the original 40 inbred lines previously and their removal from the data set did not significantly influence the hybridization results [12]. Microarray data have been deposited in the ArrayExpress database (accession: E-MTAB-639 and are also available on the DGRP website (http://dgrp.gnets.ncsu.edu/).

We analyzed array data using a Generalized Linear Model (GLM) in SAS to partition phenotypic variation between sexes (S, fixed), environments/treatments (E, fixed), the S×E interaction (fixed) and the error variance (ε). To identify environmentally responsive Class I transcripts we used an FDR<0.05 to correct for multiple tests. *Post-hoc* LSD tests were used to identify transcripts with a significant environment term. Signal intensities for those transcripts were sex-centered by subtracting the female or male mean across all conditions for each gene. Transcripts with a significant interaction term were excluded.

To resolve Class II transcripts, we applied two filters. First, we selected transcripts with cross-treatment (macroenvironmental) variance >95^th^ percentile of the macoenvironmental variance distribution of the Class I transcripts (coefficients of variation across treatments >7.06 and >7.12 for females and males, respectively). We filtered these transcripts further using an FDR>0.0001 for genetic variation among DGRP lines [12] for females and males separately. Finally, we removed overlapping transcripts between Class I and Class II. We used a form of K-means clustering (K = 2) to partition the Class II transcripts into groups of high and low expression. Specifically, for each sex we exhaustively identified the unique bipartition of Class II transcripts that minimized the total within group sum-of-squares.

The Modulated Modularity Clustering (MMC) algorithm [13] was used to group transcripts into covariant modules. Gene annotations were based on Flybase, version 5.36. Gene ontology analysis was done using the DAVID bioinformatics database, using the Benjamini correction of *p*<0.05 as criterion for enrichment [34], [35].

### Organismal Phenotypes

To measure development time, we allowed flies to lay eggs for 3 hours (10:00am–1:00pm), after which 55 eggs were collected and placed under 14 different growth conditions (300 eggs were collected to assess development time under the larval crowding condition). We counted flies, sexes separately, that eclosed every 12 hours (N = 4 vials/condition). Life span was measured by collecting three females and three males immediately 1–3 days after eclosion, transferring them to fresh vials every 2–3 days, and recording survival daily (N = 26 replicates/condition). To measure starvation stress resistance, we placed ten 3–5 days old flies in vials containing 1.5% agar, and scored survival every 8 hours (N = 4×10/sex/condition). To measure chill coma recovery, we placed 3–7 day-old flies in empty vials on ice for 3 hours, and determined their subsequent recovery time at room temperature by their ability to recover upright posture (N = 2×50 flies/sex/condition). Phenotypic data are available on the DGRP website (http://dgrp.gnets.ncsu.edu/).

### Transcript-Phenotype Associations

We used regression to identify transcripts with variation in expression levels that associated with organismal phenotypic variation (*p*<0.05). For traits with a significant sex by environment interaction, regression was applied to sexes separately (Y = μ+Exp+ε, where Exp denotes the covariate median log_2_ expression level). For traits without a significant sex by environment interaction, we used sex-centered measures of deviations from female or male means for both expression and organismal phenotypes. We used the residuals from the regressions (Y = μ+T+ε, where T denotes the trait covariate) to compute environmental correlations between transcripts significantly associated with each organismal phenotype for construction of covariant modules using MMC [13].

## Supporting Information

Figure S1 Variation of transcript abundance across 20 rearing conditions. The diagram represents 1,133 transcripts that show significant differences in expression levels across conditions. An additional 116 transcripts with a significant sex-by-environment interaction have been excluded from the diagram. Transcripts represented in the figure are sex centered. The color scale reflects the rank order of expression for individual genes across the 20 conditions with red and blue intensities indicating higher and lower expression levels, respectively. The 20 conditions from left to right, are sorted based on the number of genes with transcript levels higher than their median across conditions. Transcripts are identified in Table S1.
(PDF)

Figure S2 Examples of phenotypically plastic gene families. The diagram illustrates up-regulation or down-regulation, indicated by red and blue boxes, respectively, of members of the *Hsp*, *IM*, and *Jon* families under different treatments compared to the standard growth condition.
(PDF)

Figure S3 Pleiotropy between covariant transcriptional modules associated with four organismal phenotypes. Strength of connectivity within modules along the diagonals increases in a clockwise direction. Black lines connect modules with similar composition of covariant Class I phenotypically plastic transcripts, associated with variation in development latency, lifespan, starvation stress resistance and chill coma recovery time, shown in Figure 6. The significance of modular overlap was determined by a hypergeometric probability test with Bonferroni correction for multiple testing. Modules with fewer than three pleiotropic transcripts were not considered. Module 17 in the chill coma resistance diagram is highlighted as an example of a module that contains a large number of pleiotropic phenotypically plastic transcripts.
(PDF)

Figure S4 Distribution of genetic and environmental variation between transcript classes. (A, B) Box plots of coefficients of genetic variation (CVL) across inbred lines of Class I, Class II and robust transcripts in males (A) and females (B). (C, D) Box plots of coefficients of macroenvironmental variation (CVME) of the three transcript classes in males (C) and females (D).
(PDF)

Figure S5 Variance analysis and classification of environmentally sensitive transcripts. (A, B) Correlation structures between coefficients of within-treatment variance (CVEW) and mean transcript expression levels across all treatments for males (A) and females (B). (C, D) Correlation structures between standard deviations of the coefficients of within-treatment variance (STD_CVEW) and mean transcript expression levels across all treatments in males (C) and females (D). Class I transcripts have significant lower within-treatment variation than Class II transcripts. Transcripts associated with correlation structures that are not explained by Class II transcripts are also distinct from Class I transcripts with higher within-treatment variation (CVEW) and variance of the within-treatment variations (STD_CVEW). (E, F) Relationships between coefficients of within-line variation (CVE) and mean transcript expression levels across all treatments in males (E) and females (F). (G, H) Correlations between mean transcript expression across the original 40 inbred lines and the mean transcript expression across the 20 treatments of the reconstituted outbred population in males (G) and females (H). Red dots indicate Class I transcripts, green and purple dots indicate Class II transcripts in males (A, C, E, G) and females (B, D, F, H), respectively, and grey dots indicate robust transcripts.
(PDF)

Figure S6 Relationship between sexual dimorphism for the mean and coefficient of macroenvironmental variance of gene expression. (A) All Class I phenotypically plastic transcripts. (B) All Class II phenotypically plastic transcripts. (C) A random sample of 1,500 robust transcripts.
(PDF)

Figure S7 Distribution of ω (d_N_/d_S_), using 6 outgroup species [21]–[22]. Both Class I and Class II transcripts have significantly different distributions of ω from that of the robust transcripts (G = 34.33, *p*<0.0001, and G = 334.48, *p*<0.00001, respectively), which is consistent with the result shown in Figure 8.
(PDF)

Figure S8 Distribution of expression patterns of modules associated with development time, life span, starvation resistance and chill coma recovery time. Tissue specific expression patterns of modules associated with (A) development time, (B) life span, (C) starvation resistance, and (D) chill coma recovery time were analyzed using the FlyAtlas database [30]. For all organismal phenotypes, correlated transcripts are generally expressed in multiple, but not all tissues. Expression in testes or ovaries is observed only infrequently, whereas expression in the digestive tract (*e.g.* both the larval and adult midgut) is prominent. Expression of some modules is observed in spermatheca. Similarly, the fat body features as a prominent organ for expression of Class I covariant transcripts. Some modules show enriched expression in brain, heart and salivary gland. Modules that comprise transcripts associated with development (Figure 6A) are frequently expressed in brain, whereas enrichment in brain is less evident for the other traits. Module 8, associated with development time, is enriched exclusively in larval trachea under the standard growth condition, but shows differential expression under various environmental challenges in adults.
(PDF)

Table S1 Quantitative genetic analyses of variation for 14,400 expressed transcripts of the outbred population across 20 treatments. Expression is measured as the median log2 intensity of PM transcripts in each probe set. Class indicates the Class I and Class II phenotypically plastic transcripts. FDR is False Discovery Rate, CV is the coefficient of variation, CVME is the coefficient of cross treatment (macroenvironmental) variance, CVEW is the mean coefficient of within treatment variation, and STD_CVEW is the standard deviation of coefficient of within treatment variation. Std_Mean is the standard deviation of treatment mean. Inbred line means, genetic variation among lines (CVL) and micro-environmental variation within lines (CVE) are adopted from (12). MMC (13) revealed 103 modules of 1,133 Class I transcripts. |r| is the average correlation of each variable transcript with all other variable transcripts.
(XLSX)

Table S2 Pair-wise comparisons of the phenotypically plastic Class I transcripts under different treatments with the standard growth condition.
(XLSX)

Table S3 Gene ontology analysis of Class I phenotypically plastic transcripts using DAVID*.
(XLSX)

Table S4 Class I phenotypically plastic transcripts associated with development time and clustered into modules using MMC (13).
(XLSX)

Table S5 Class I phenotypically plastic transcripts associated with life span and clustered into modules using MMC (13).
(XLSX)

Table S6 Class I phenotypically plastic transcripts associated with chill coma and clustered into modules using MMC (13).
(XLSX)

Table S7 Class I phenotypically plastic transcripts associated with starvation resistance and clustered into modules using MMC (13).
(XLSX)

Table S8 Gene ontology analysis of overlapping Class II transcripts between high expressed transcripts in females and low expressed transcripts in males using DAVID (34).
(XLSX)

Table S9 Gene ontology analysis of overlapping Class II transcripts between low expressed transcripts in females and high expressed transcripts in males using DAVID (34).
(XLSX)

Table S10 Gene ontology analysis of female specific Class II transcripts using DAVID (34).
(XLSX)

Table S11 Gene ontology analysis of male specific Class II transcripts using DAVID (34).
(XLSX)

## References

[1] Bradshaw AD (1965). Evolutionary significance of phenotypic plasticity in plants.. Advances in Genetics.

[2] Waddington CH (1942). Canalization of developmnet and the inheritance of acquired characters.. Nature.

[3] Waddington CH (1959). Canalization of development and genetic assimilation of acquired characters.. Nature.

[4] Gibson G (2009). Decanalization and the origin of complex disease.. Nat Rev Genet.

[5] Lee CK, Weindruch R, Prolla TA (2000). Gene-expression profile of the ageing brain in mice.. Nat Genet.

[6] Zou S, Meadows S, Sharp L, Jan LY, Jan YN (2000). Genome-wide study of aging and oxidative stress response in *Drosophila melanogaster*.. Proc Natl Acad Sci U S A.

[7] Cao SX, Dhahbi JM, Mote PL, Spindler SR (2001). Genomic profiling of short- and long-term caloric restriction effects in the liver of aging mice.. Proc Natl Acad Sci U S A.

[8] Pletcher SD, Macdonald SJ, Marguerie R, Certa U, Stearns SC (2002). Genome-wide transcript profiles in aging and calorically restricted *Drosophila melanogaster*.. Curr Biol.

[9] Sambandan D, Carbone MA, Anholt RRH, Mackay TFC (2008). Phenotypic plasticity and genotype by environment interaction for olfactory behavior in *Drosophila melanogaster*.. Genetics.

[10] Vinuela A, Snoek LB, Riksen JA, Kammenga JE (2010). Genome-wide gene expression regulation as a function of genotype and age in *C. elegans*.. Genome Res.

[11] Levine MT, Eckert ML, Begun DJ (2011). Whole-genome expression plasticity across tropical and temperate *Drosophila melanogaster* populations from Eastern Australia.. Mol Biol Evol.

[12] Ayroles JF, Carbone MA, Stone EA, Jordan KW, Lyman RF (2009). Systems genetics of complex traits in *Drosophila melanogaster*.. Nat Genet.

[13] Stone EA, Ayroles JF (2009). Modulated modularity clustering as an exploratory tool for functional genomic inference.. PLoS Genet.

[14] Bushati N, Cohen SM (2007). microRNA functions.. Annu Rev Cell Dev Biol.

[15] Roy S, Ernst J, Kharchenko PV, Kheradpour P, Negre N (2010). Identification of functional elements and regulatory circuits by Drosophila modENCODE.. Science.

[16] Sambucetti P, Loeschcke V, Norry FM (2006). Developmental time and size-related traits in *Drosophila buzzatii* along an altitudinal gradient from Argentina.. Hereditas.

[17] Mensch J, Lavagnino N, Carreira VP, Massaldi A, Hasson E (2008). Identifying candidate genes affecting developmental time in *Drosophila melanogaster*: pervasive pleiotropy and gene-by-environment interaction.. BMC Dev Biol.

[18] Chapman T, Liddle LF, Kalb JM, Wolfner MF, Partridge L (1995). Cost of mating in *Drosophila melanogaster* females is mediated by male accessory gland products.. Nature.

[19] Sohal RS, Weindruch R (1996). Oxidative stress, caloric restriction, and aging.. Science.

[20] Fontana L, Partridge L, Longo VD (2010). Extending healthy life span–from yeast to humans.. Science.

[21] Clark AG, Eisen MB, Smith DR, Bergman CM, Oliver B (2007). Evolution of genes and genomes on the *Drosophila* phylogeny.. Nature.

[22] Larracuente AM, Sackton TB, Greenberg AJ, Wong A, Singh ND (2008). Evolution of protein-coding genes in *Drosophila*.. Trends Genet.

[23] Mackay TFC, Richards S, Stone EA, Barbadilla A, Ayroles JF (2012). The *Drosophila melanogaster* Genetic Reference Panel.. Nature.

[24] Zhou S, Stone EA, Mackay TFC, Anholt RRH (2009). Plasticity of the chemoreceptor repertoire in *Drosophila melanogaster*.. PLoS Genet.

[25] Strode C, Wondji CS, David JP, Hawkes NJ, Lumjuan N (2008). Genomic analysis of detoxification genes in the mosquito *Aedes aegypti*.. Insect Biochem Mol Biol.

[26] Feyereisen R (2006). Evolution of insect P450.. Biochem Soc Trans.

[27] Claudianos C, Ranson H, Johnson RM, Biswas S, Schuler MA (2006). A deficit of detoxification enzymes: pesticide sensitivity and environmental response in the honeybee.. Insect Mol Biol.

[28] Richards S, Gibbs RA, Weinstock GM, Brown SJ, Denell R (2008). The genome of the model beetle and pest *Tribolium castaneum*.. Nature.

[29] Ranson H, Claudianos C, Ortelli F, Abgrall C, Hemingway J (2002). Evolution of supergene families associated with insecticide resistance.. Science.

[30] Chintapalli VR, Wang J, Dow JA (2007). Using FlyAtlas to identify better *Drosophila melanogaster* models of human disease.. Nat Genet.

[31] Zhang XS, Hill WG (2007). Competition can maintain genetic but not environmental variance in the presence of stabilizing selection.. Evolution.

[32] Via S, Gomulkiewicz R, De Jong G, Scheiner SM, Schlichting CD (1995). Adaptive phenotypic plasticity: consensus and controversy.. Trends Ecol Evol.

[33] Mackay TFC, Stone EA, Ayroles JF (2009). The genetics of quantitative traits: challenges and prospects.. Nat Rev Genet.

[34] Huang da W, Sherman BT, Lempicki RA (2009). Systematic and integrative analysis of large gene lists using DAVID bioinformatics resources.. Nat Protoc.

[35] Huang da W, Sherman BT, Lempicki RA (2009). Bioinformatics enrichment tools: paths toward the comprehensive functional analysis of large gene lists.. Nucleic Acids Res.

[36] Yu J, Pacifico S, Liu G, Finley RL (2008). DroID: the *Drosophila* Interactions Database, a comprehensive resource for annotated gene and protein interactions.. BMC Genomics.

[37] Murali T, Pacifico S, Yu J, Guest S, Roberts GG (2011). DroID 2011: a comprehensive, integrated resource for protein, transcription factor, RNA and gene interactions for *Drosophila*.. Nucleic Acids Res.

