# Supplementary figures and images for: Phenotypic Plasticity of the Drosophila Transcriptome
Source: PLoS Genet. 2012 Mar 29;8(3):e1002593. doi: 10.1371/journal.pgen.1002593 (PMC3315458; doi:10.1371/journal.pgen.1002593)

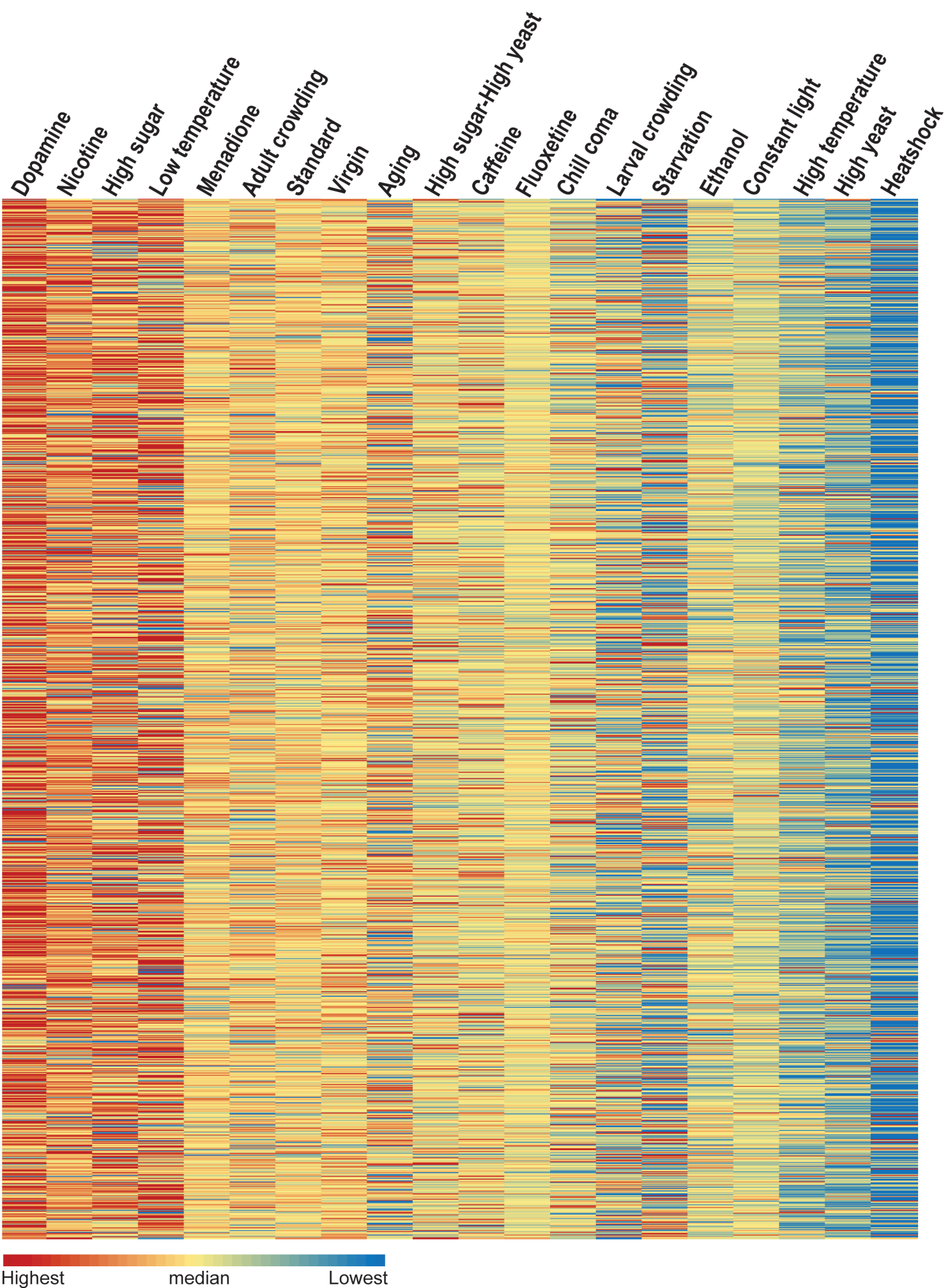

Supplement: Figure S1 — Variation of transcript abundance across 20 rearing conditions. The diagram represents 1,133 transcripts that show significant differences in expression levels across conditions. An additional 116 transcripts with a significant sex-by-environment interaction have been excluded from the diagram. Transcripts represented in the figure are sex centered. The color scale reflects the rank order of expression for individual genes across the 20 conditions with red and blue intensities indicating higher and lower expression levels, respectively. The 20 conditions from left to right, are sorted based on the number of genes with transcript levels higher than their median across conditions. Transcripts are identified in Table S1. (PDF) [file pgen.1002593.s001.pdf]

Chill coma resistance

Development time

17

Life span

Starvation resistance

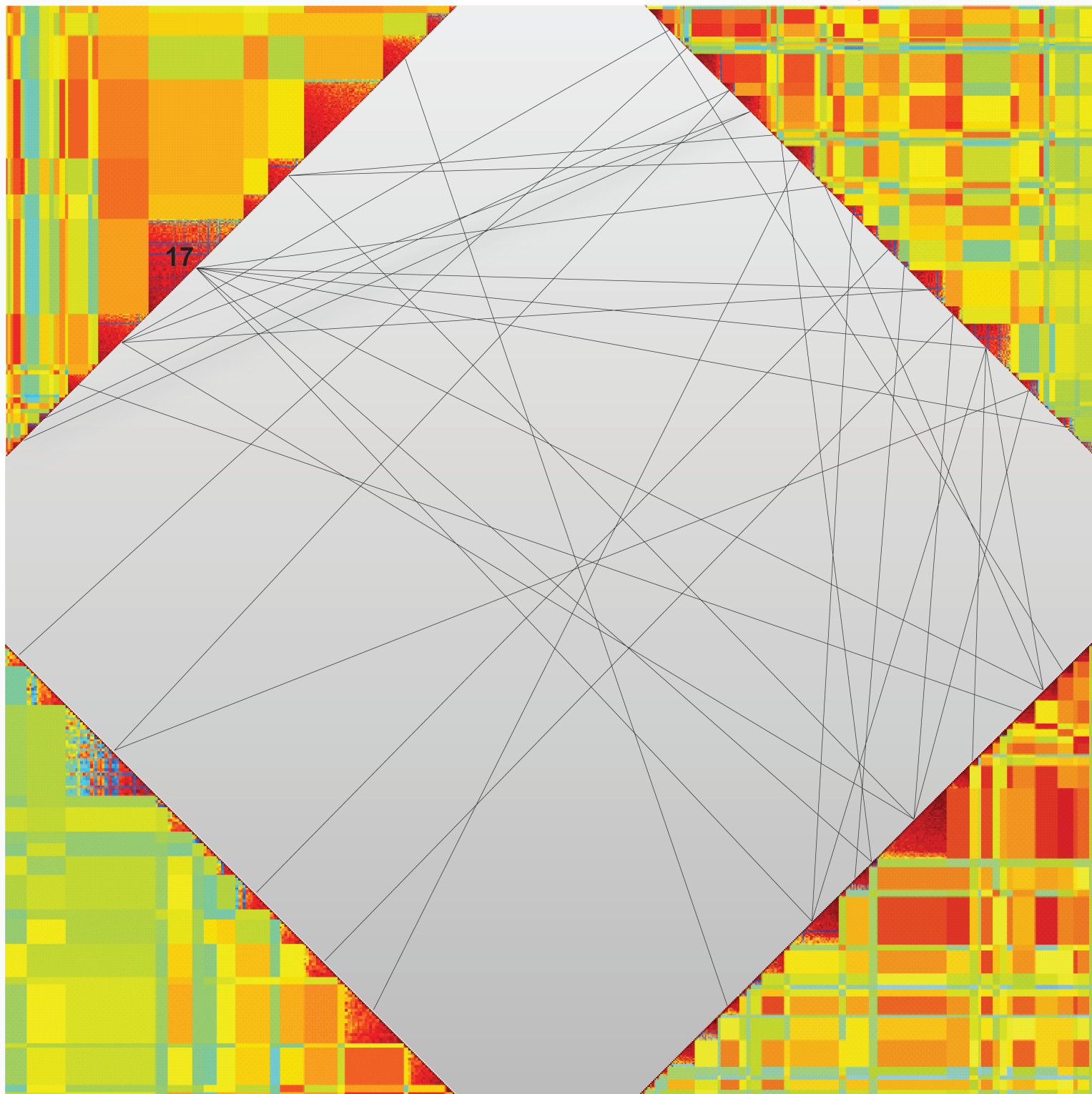

Supplement: Figure S3 — Pleiotropy between covariant transcriptional modules associated with four organismal phenotypes. Strength of connectivity within modules along the diagonals increases in a clockwise direction. Black lines connect modules with similar composition of covariant Class I phenotypically plastic transcripts, associated with variation in development latency, lifespan, starvation stress resistance and chill coma recovery time, shown in Figure 6. The significance of modular overlap was determined by a hypergeometric probability test with Bonferroni correction for multiple testing. Modules with fewer than three pleiotropic transcripts were not considered. Module 17 in the chill coma resistance diagram is highlighted as an example of a module that contains a large number of pleiotropic phenotypically plastic transcripts. (PDF) [file pgen.1002593.s003.pdf]

## Male

**A**

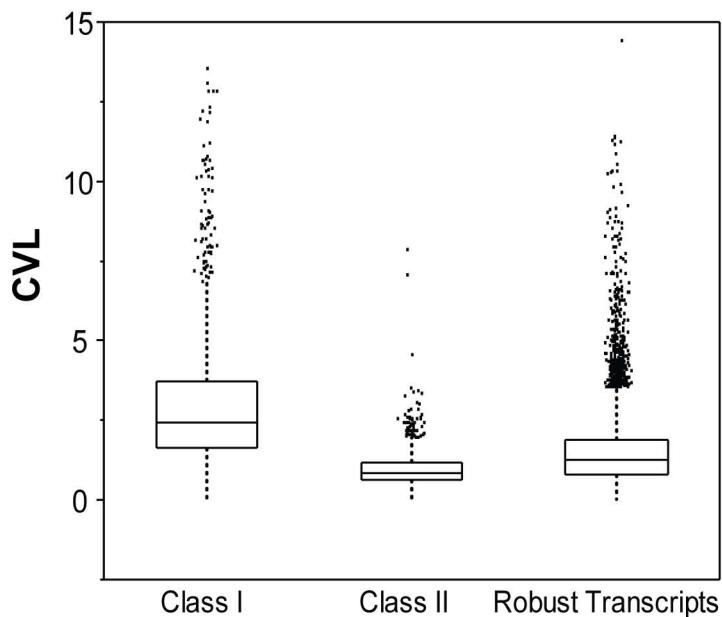

## Female

**B**

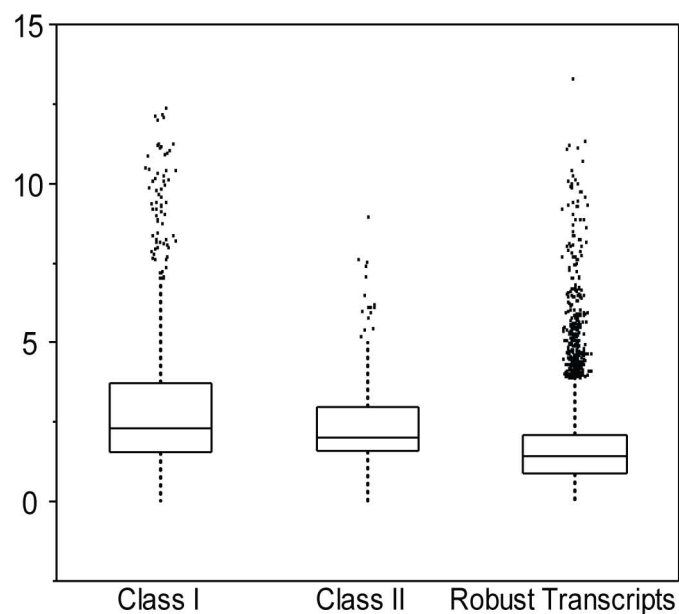

**C**

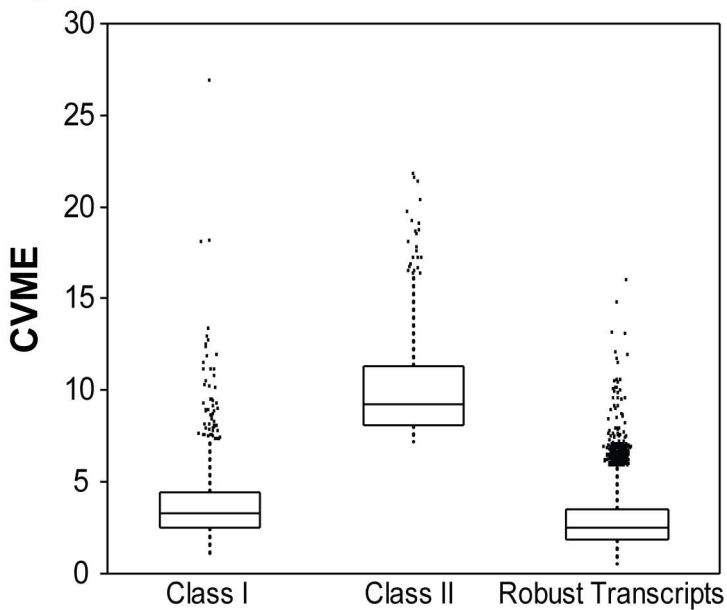

**D**

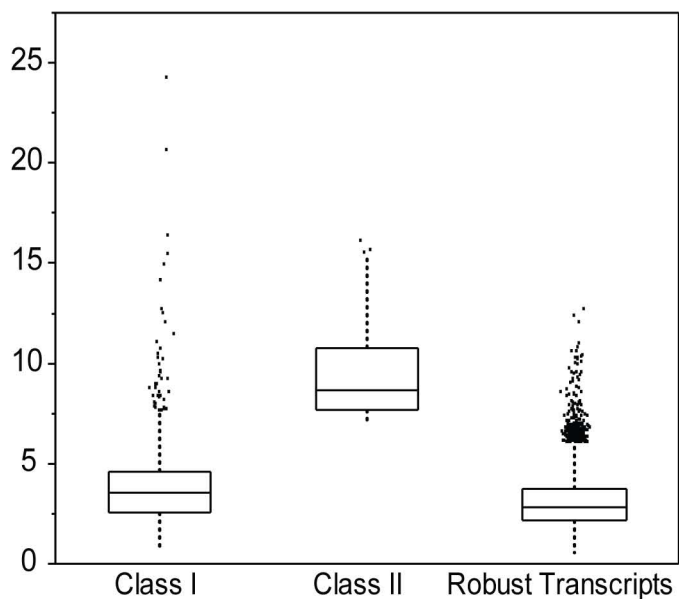

Supplement: Figure S4 — Distribution of genetic and environmental variation between transcript classes. (A, B) Box plots of coefficients of genetic variation (CVL) across inbred lines of Class I, Class II and robust transcripts in males (A) and females (B). (C, D) Box plots of coefficients of macroenvironmental variation (CVME) of the three transcript classes in males (C) and females (D). (PDF) [file pgen.1002593.s004.pdf]

## Male

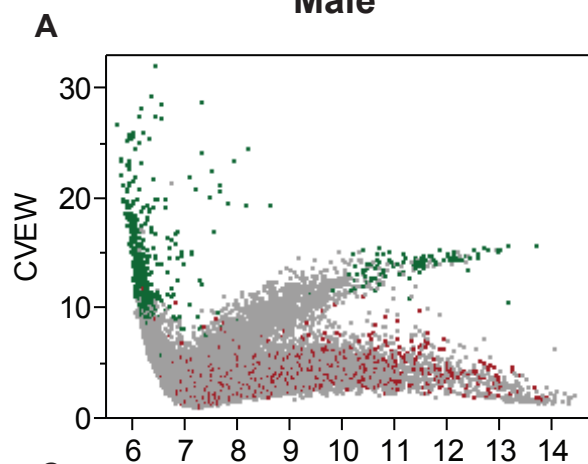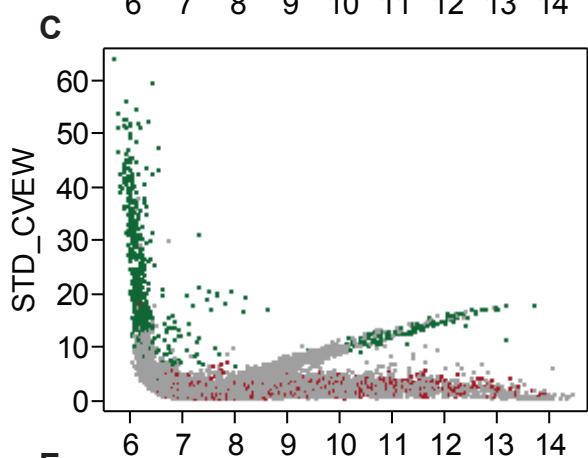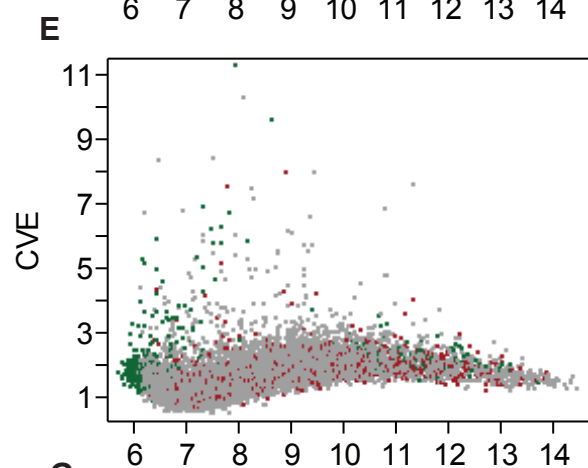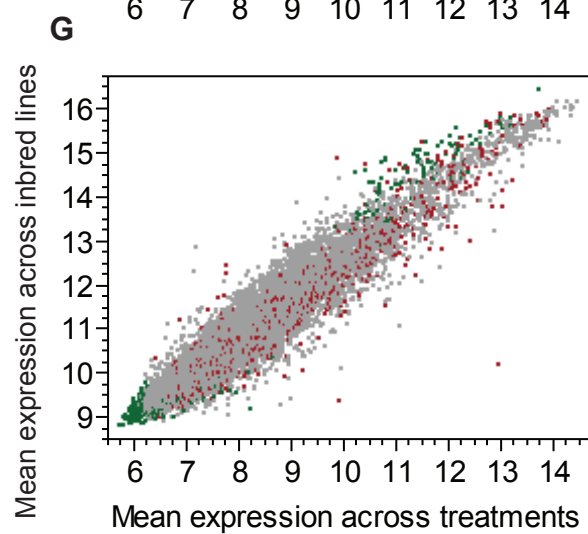

## Female

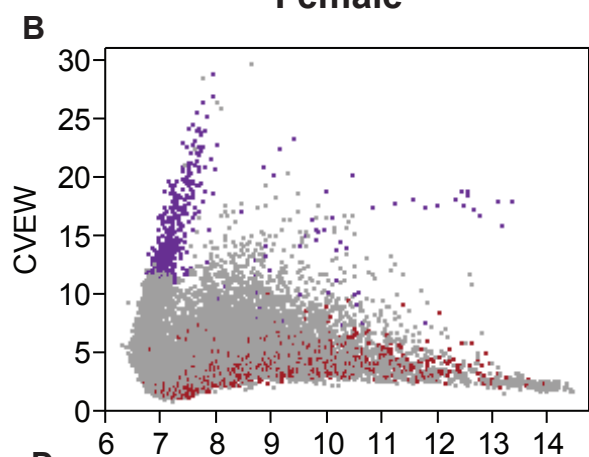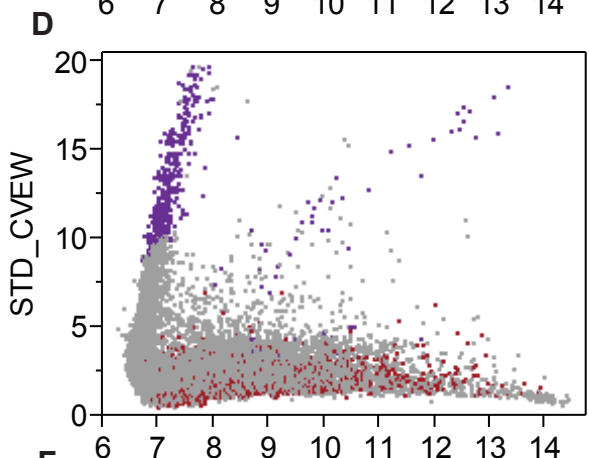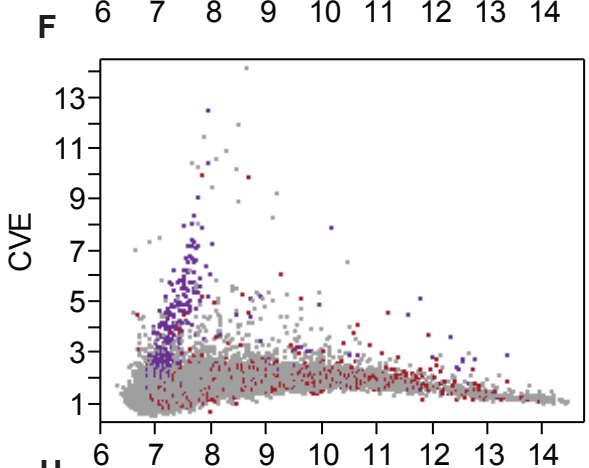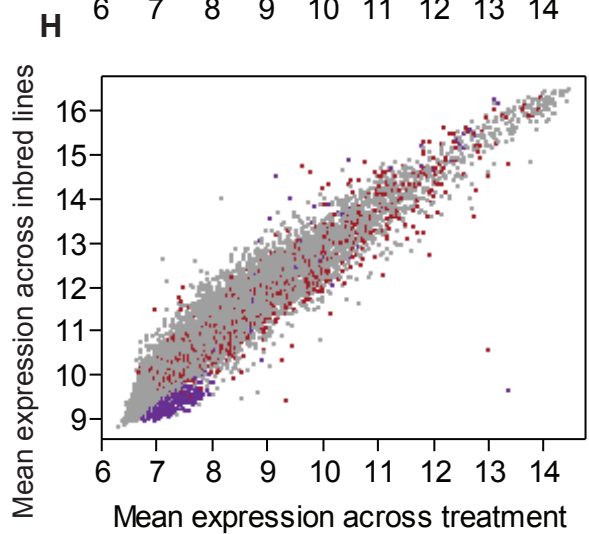

Supplement: Figure S5 — Variance analysis and classification of environmentally sensitive transcripts. (A, B) Correlation structures between coefficients of within-treatment variance (CVEW) and mean transcript expression levels across all treatments for males (A) and females (B). (C, D) Correlation structures between standard deviations of the coefficients of within-treatment variance (STD_CVEW) and mean transcript expression levels across all treatments in males (C) and females (D). Class I transcripts have significant lower within-treatment variation than Class II transcripts. Transcripts associated with correlation structures that are not explained by Class II transcripts are also distinct from Class I transcripts with higher within-treatment variation (CVEW) and variance of the within-treatment variations (STD_CVEW). (E, F) Relationships between coefficients of within-line variation (CVE) and mean transcript expression levels across all treatments in males (E) and females (F). (G, H) Correlations between mean transcript expression across the original 40 inbred lines and the mean transcript expression across the 20 treatments of the reconstituted outbred population in males (G) and females (H). Red dots indicate Class I transcripts, green and purple dots indicate Class II transcripts in males (A, C, E, G) and females (B, D, F, H), respectively, and grey dots indicate robust transcripts. (PDF) [file pgen.1002593.s005.pdf]

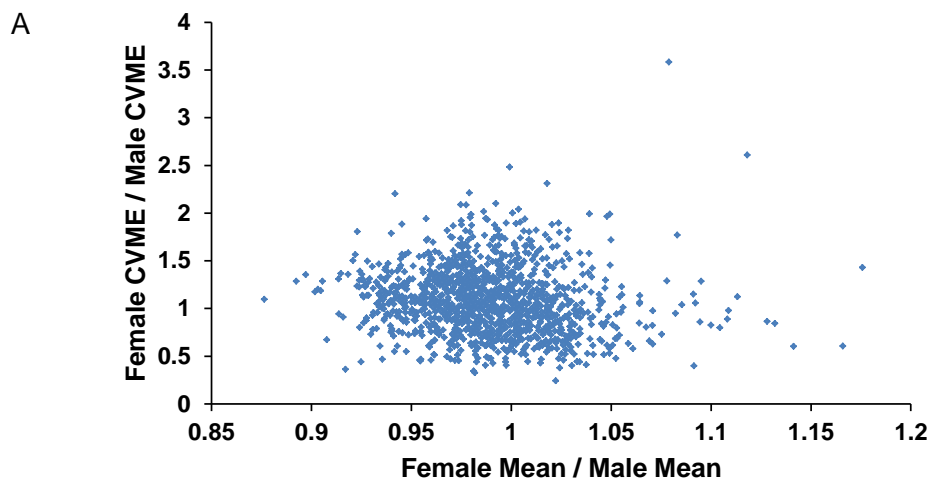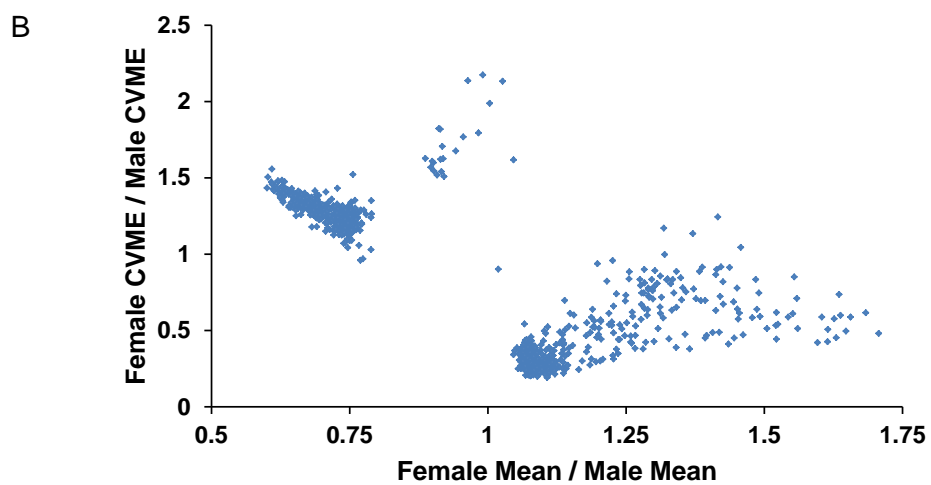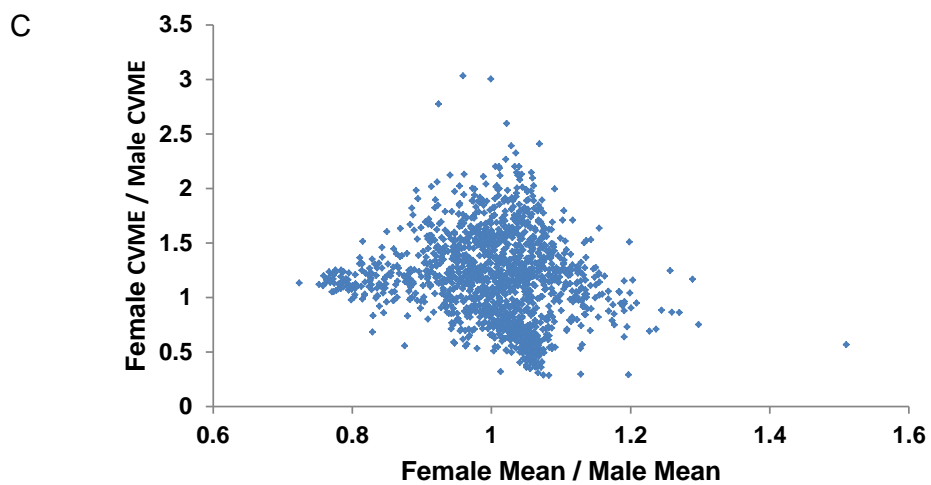

Supplement: Figure S6 — Relationship between sexual dimorphism for the mean and coefficient of macroenvironmental variance of gene expression. (A) All Class I phenotypically plastic transcripts. (B) All Class II phenotypically plastic transcripts. (C) A random sample of 1,500 robust transcripts. (PDF) [file pgen.1002593.s006.pdf]

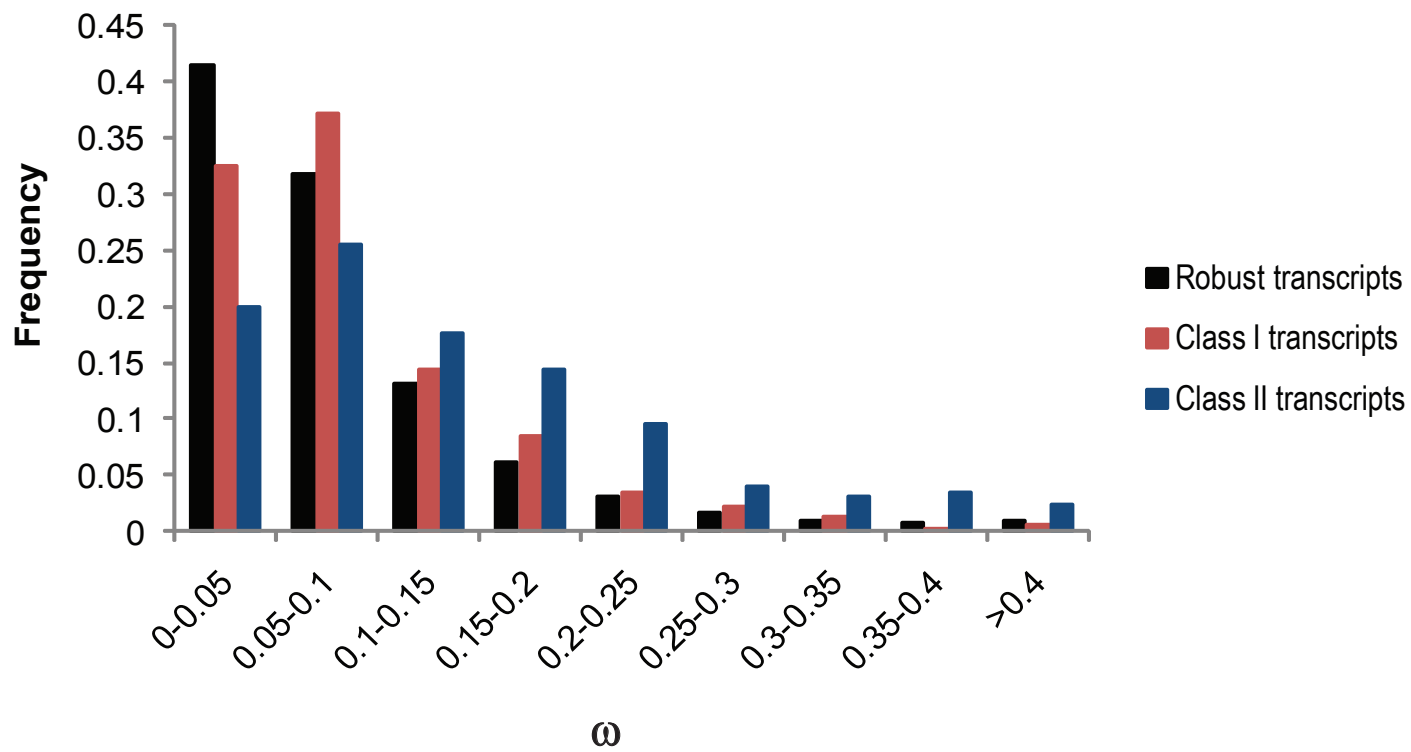

Supplement: Figure S7 — Distribution of ω (dN/dS), using 6 outgroup species [21]–[22]. Both Class I and Class II transcripts have significantly different distributions of ω from that of the robust transcripts (G = 34.33, p<0.0001, and G = 334.48, p<0.00001, respectively), which is consistent with the result shown in Figure 8. (PDF) [file pgen.1002593.s007.pdf]

## A Development time

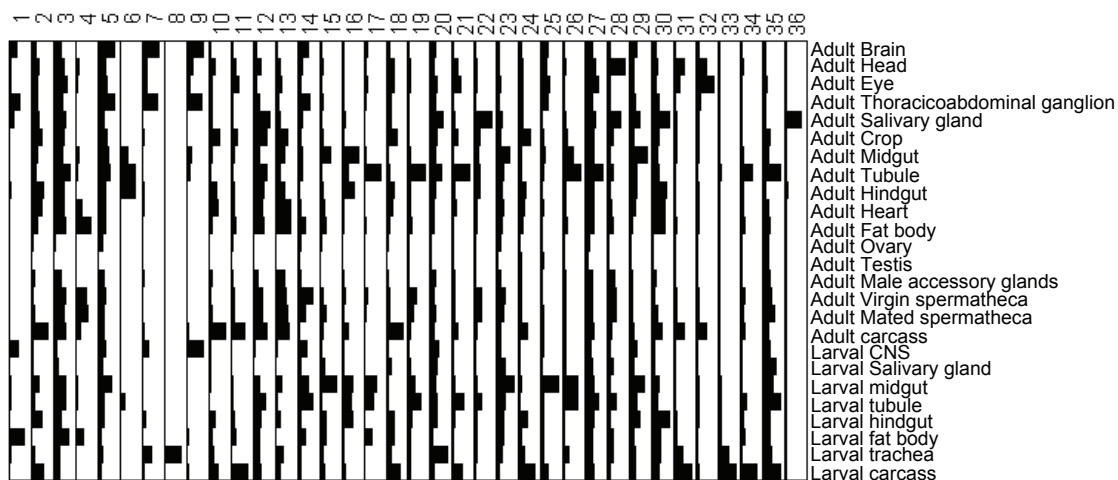

## B Life span

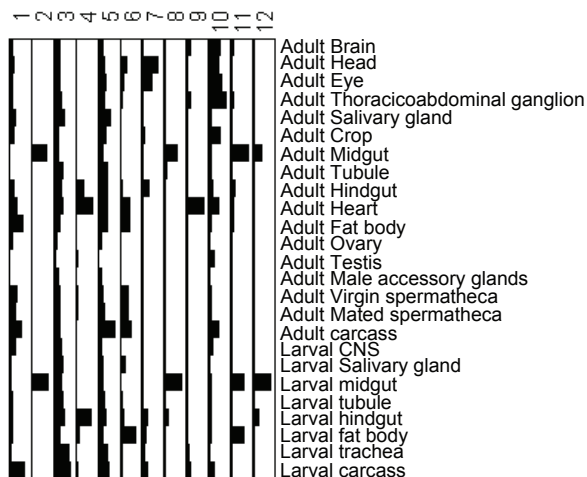

## C Starvation resistance

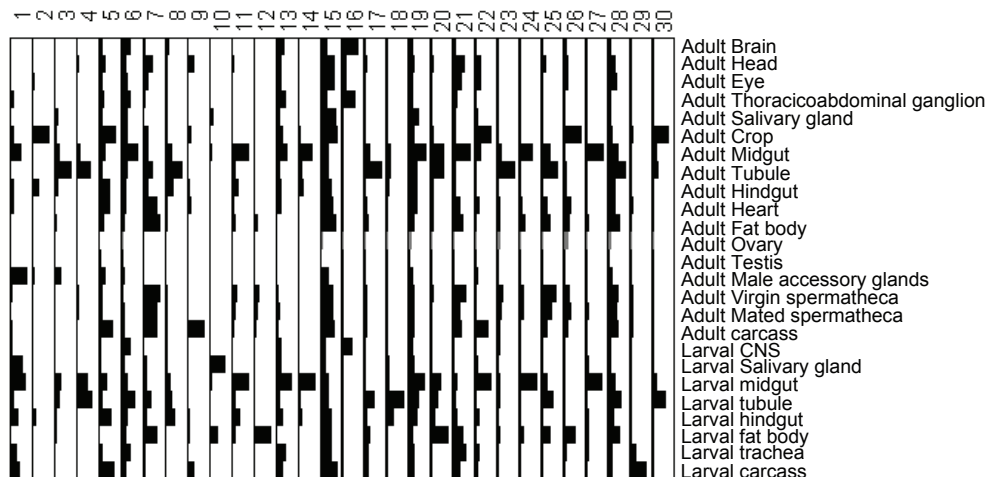

## D Chill Coma Recovery

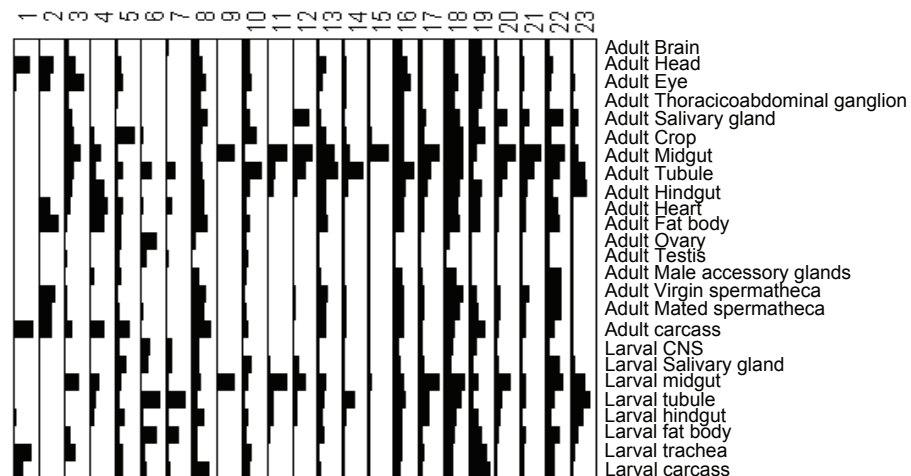

Supplement: Figure S8 — Distribution of expression patterns of modules associated with development time, life span, starvation resistance and chill coma recovery time. Tissue specific expression patterns of modules associated with (A) development time, (B) life span, (C) starvation resistance, and (D) chill coma recovery time were analyzed using the FlyAtlas database [30]. For all organismal phenotypes, correlated transcripts are generally expressed in multiple, but not all tissues. Expression in testes or ovaries is observed only infrequently, whereas expression in the digestive tract (e.g. both the larval and adult midgut) is prominent. Expression of some modules is observed in spermatheca. Similarly, the fat body features as a prominent organ for expression of Class I covariant transcripts. Some modules show enriched expression in brain, heart and salivary gland. Modules that comprise transcripts associated with development (Figure 6A) are frequently expressed in brain, whereas enrichment in brain is less evident for the other traits. Module 8, associated with development time, is enriched exclusively in larval trachea under the standard growth condition, but shows differential expression under various environmental challenges in adults. (PDF) [file pgen.1002593.s008.pdf]
