# Supplementary material for: Phenotypic Plasticity of the Drosophila Transcriptome
Source: PLoS Genet. 2012 Mar 29;8(3):e1002593. doi: 10.1371/journal.pgen.1002593 (PMC3315458; doi:10.1371/journal.pgen.1002593)
Supplement: Figure S2 — Examples of phenotypically plastic gene families. The diagram illustrates up-regulation or down-regulation, indicated by red and blue boxes, respectively, of members of the Hsp, IM, and Jon families under different treatments compared to the standard growth condition. (PDF) [file pgen.1002593.s002.pdf]

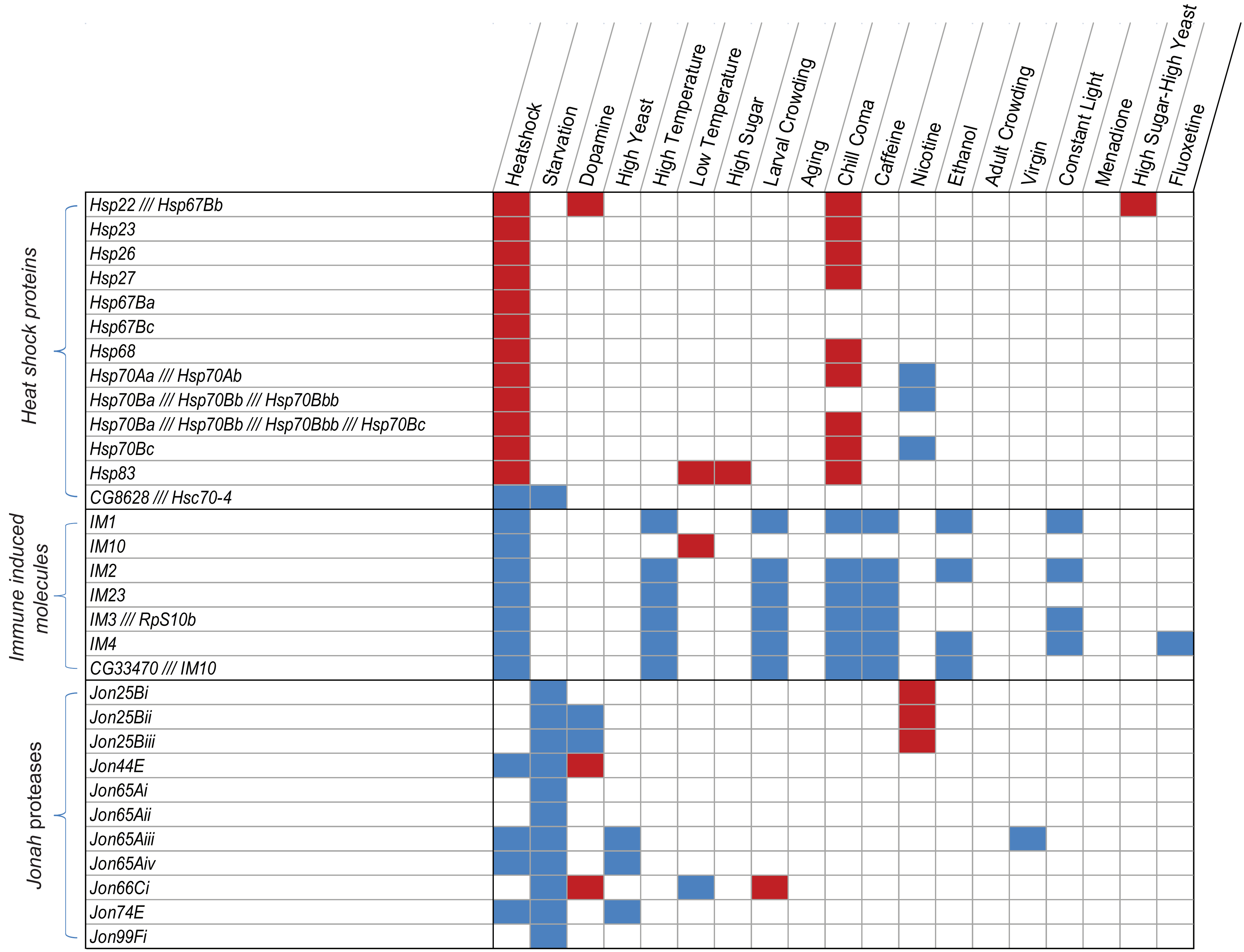
Heatshock
Starvation
Dopamine
High Yeast
High Temperature
Low Temperature
High Sugar
Larval Crowding
Aging
Chill Coma
Caffeine
Nicotine
Ethanol
Adult Crowding
Virgin
Constant Light
Menadione
High Sugar-High Yeast
Fluoxetine
Heat shock proteins
Hsp22 /// Hsp67Bb
Hsp23
Hsp26
Hsp27
Hsp67Ba
Hsp67Bc
Hsp68
Hsp70Aa /// Hsp70Ab
Hsp70Ba /// Hsp70Bb /// Hsp70Bbb
Hsp70Ba /// Hsp70Bb /// Hsp70Bbb /// Hsp70Bc
Hsp70Bc
Hsp83
CG8628 /// Hsc70-4
Immune induced molecules
IM1
IM10
IM2
IM23
IM3 /// RpS10b
IM4
CG33470 /// IM10
Jonah proteases
Jon25Bi
Jon25Bii
Jon25Biii
Jon44E
Jon65Ai
Jon65Aii
Jon65Aiii
Jon65Aiv
Jon66Ci
Jon74E
Jon99Fi
